# Allelic variation in a cluster of epsilon glutathione S-transferase genes contributes to DDT and pyrethroid resistance in the major African malaria vector *Anopheles funestus*

**DOI:** 10.1186/s12864-025-11637-3

**Published:** 2025-05-07

**Authors:** Mersimine F. M. Kouamo, Sulaiman S. Ibrahim, Abdullahi Muhammad, Mahamat Gadji, Jack Hearn, Charles S. Wondji

**Affiliations:** 1 Centre for Research in Infectious Diseases (CRID), P.O. Box 13591, Yaoundé, Cameroon; 2 Department of Biochemistry, Bayero University, PMB, 3011, Kano, Nigeria; 3 Vector Biology Department, Liverpool School of Tropical Medicine, Pembroke Place, Liverpool, L3 5QA UK; 4 Department of Microbiology, Faculty of Science, University of Yaoundé I, P.O. Box 812, Yaoundé, Cameroon; 5 Centre for Epidemiology and Planetary Health, School of Veterinary Medicine, Scotland’s Rural College Inverness IV2 5NA, Inverness, UK

**Keywords:** Malaria, *Anopheles funestus*, pyrethroids/DDT, Metabolic resistance, Glutathione S-transferase, Transgenic expression, metabolic assay

## Abstract

**Background:**

Insecticide resistance in malaria vectors is a serious challenge to malaria control and elimination. Elucidation of the role of detoxification genes in resistance is necessary to develop targeted strategies to reduce malaria burden. Glutathione S-transferase epsilon clusters (*GSTe* genes) are upregulated in DDT- and pyrethroid-resistant *Anopheles funestus* mosquitoes across Africa. However, except for *GSTe2*, the molecular mechanisms behind this upregulation remain unclear. Here, we established that overexpression and allelic variation of GSTe genes contribute to insecticide resistance in African malaria vector *An. funestus* s.s.

**Methods:**

Transcriptomic and genomic analyses of *GST*e genes were conducted, followed by in silico structural analysis, and functional characterization of *GSTe3*, *GSTe4* and *GSTe6* using metabolic assay and transgenic expression in *Drosophila* flies.

**Results:**

Transcriptomic and genomic analyses reveal changes in gene expression and genetic diversity of *GSTes* cluster in *An. funestus* across Africa. Cloning of cDNAs of *GST*es from different regions of Africa detected allelic variants under selection, including A^17^D^26^T^158^-GSTe3, L^135^H^191^A^189^-GSTe4 in West/Central Africa, and T^169^S^201^ E^210^-*GST*e6 present only in West/Southern Africa. Furthermore, in silico analysis of BN-GSTe3, MWI-GSTe3, BN-GSTe4, MWI-GSTe4, CMR-GSTe6 and, BN-GSTe6 alleles revealed that allelic variations increase the binding cavity in the active site of these *GST*es with stronger affinities observed towards DDT and permethrin. All recombinant *GST*es significantly metabolize DDT (41–63%) and permethrin (13–25%). Additionally, BN-GSTe4 (L^135^H^191^A^189^-GSTe4) variant significantly metabolizes deltamethrin (28.75%), compared to the wild-type allele (15.99%; *p* < 0.05). Transgenic expression of the *GST*es in *Drosophila melanogaster* flies revealed reduced DDT mortalities in flies expressing the selected alleles (39–55%; p˂0.001), compared to control group (98%). Similar resistance patterns were observed toward permethrin and deltamethrin.

**Conclusion:**

These findings established the role of *GST*es in conferring cross-resistance to pyrethroids and DDT, highlighting the role of these genes in metabolic resistance in *An. funestus*, which complicates malaria control using the above key insecticides.

**Supplementary Information:**

The online version contains supplementary material available at 10.1186/s12864-025-11637-3.

## Introduction

Malaria remains one of the major debilitating vector-borne diseases in the tropical world, and notably in Africa. According to recent reports published by the World Health Organization (WHO), 249 million cases of malaria were recorded worldwide in 2023 with more than 608,000 deaths [1]. Africa still records 95% of the global burden, with children under the age of 5 and pregnant women the main victims. Over the past decade, remarkable progress has been made in reducing malaria morbidity and mortality [2]. However, progress in reducing the disease burden has slowed down due among other factors to the growing and multiple resistance to vector control insecticides [3–5]. While the fight against malaria vectors focuses on the use of insecticides, reliance on these chemicals inevitably select for resistance in mosquitoes [6–8]. The major malaria vector *Anopheles funestus s.s.* has developed resistance to most classes of insecticides, notably pyrethroids, organochlorines, and carbamates [9, 10]. Recent studies have highlighted the association between high levels of insecticide resistance and a significant loss of efficacy of vector control tools including synergist-based nets such as piperonyl butoxide (PBO) in *An. funestus* populations [11–13]. Consequently, insecticide resistance is a major threat to the continued effectiveness of vector control tools [14] and is leading to an increase in the number of malaria cases since 2016 as reported by the WHO [15, 16]. A key step in resistance management is to understand the genetic basis of insecticide resistance and develop tools to anticipate and monitor the spread of this resistance in the field.

Two main mechanisms of insecticide resistance have been identified in Anopheles mosquitoes: target site mutations, such as the *kdr* and *ace* mutations which confer insensitivity to pyrethroids/DDT and carbamate/organophosphate insecticides, respectively [17, 18]; and metabolic resistance orchestrated by detoxification enzymes such as cytochrome P450s (CYP450s), glutathione S-transferases (*GSTs*) and carboxylesterases [12, 19–21]. Beside overexpression of the metabolic genes, the molecular basis of metabolic resistance is complex and includes other mechanisms such as key amino acid changes that alter protein structure and/or function (allelic variation) [22, 23]. Previous studies have shown that allelic variation in cytochrome P450s *CYP6P9a* and *CYP6P9b* [24], and the presence of genomic structural variations such as a 6.5 kb insertion in southern Africa [25] as well as a 4.3 kb transposon-containing structural variant in Central-East Africa [26], induce resistance to insecticides, reducing efficacy of bed nets in *An. funestus*. Similarly, a single amino acid change in the cytochrome *Cyp6g1* has been reported to confer resistance to pyrethroids in *D. melanogaster* [27, 28]. Furthermore, it was demonstrated that the presence of L119F-GSTe2 mutation in *An. funestus* not only confer DDT and permethrin resistance [29, 30], but is also associated with increased malaria transmission [31] despite the presence of a fitness cost in *An. funestus* [32, 33].

Previous RNAseq-based transcriptomic studies in *An. funestus* have highlighted the up-regulation of several *GST epsilon* genes in populations from different regions in Africa, albeit with variable fold-changes [34]. However, the molecular basis of this up-regulation remains uncharacterized and there is still no evidence of their metabolic ability in detoxifying insecticides apart from *GSTe2* [29].

The present study aims to address this knowledge gap by deciphering the genetic factors driving the up-regulation of the cluster of *GST epsilon* genes and to functionally establish their metabolic activity on a range of insecticides in *An. funestus* to confirm their role in the observed resistance. Here, we investigated the genetic diversity of the *GSTe* genes cluster across Africa and identified allelic variants potentially associated with insecticide resistance. We then assessed the impact of *GSTe* genes cluster expression and the contribution of *GSTe3*, *GSTe4*, and *GSTe6* allelic variants to insecticide resistance. This included in silico structural characterization to assess how allelic variation affects insecticide affinity, and metabolism activity assays to measure the effect of recombinantly expressed *GSTe* proteins against DDT and pyrethroid insecticides. In addition, we investigated whether expression of the above *GSTe* alleles alone could confer insecticide resistance using transgenic *Drosophila melanogaster* flies. This study demonstrate that *GST* epsilon genes are significantly contributing to DDT/pyrethroid resistance through allelic variation and over-transcription.

## Materials and methods

### Genetic diversity of glutathione S-transferase epsilon (*GSTes*) genes cluster across Africa

A comparative transcriptomic analysis of the eight *GSTe* genes cluster (*GSTe1*, *GSTe2*, *GSTe3*, *GSTe4*, *GSTe5*, *GSTe6*, *GSTe7* and *GSTe8*) was conducted across five African countries: Benin and Ghana (West Africa), Cameroon (Central Africa), Malawi (Southern Africa), and Uganda (East Africa) to examine their contribution to DDT and pyrethroid resistance in *An. funestus*. The study also used RNA-Seq data from 2022, analysed using the methodology previously described [35]. Comparative analyses were performed using female mosquitoes unexposed to any insecticide (i), F_1_ mosquitoes alive following DDT exposure (ii), relative to the FANG - a fully susceptible lab colony, (iii) to evaluate the contemporary expression profiles of the *GSTe* genes. Differentially expressed genes (DEGs) were identified by comparing the transcriptomes of these mosquitoes to that of FANG using DESeq2 [36], with overexpressed genes defined as those having a corrected *p*-value < 0.05 and a log_2_ fold change > 2.

In addition to transcriptomic profiling, genetic differentiation and selection analyses were conducted across these populations using Genome-Wide Association Studies (GWAS) of current PoolSeq data and individual whole-genome sequencing (iWGS) data from the multicountry MalariaGEN project (https://www.malariagen.net/project/anopheles-funestus-genomic-surveillance-project/*).* A windowed *F*_*ST*_ analysis of chromosome 2 L, where the *GSTes* locus is located, was conducted using PoolSeq data collected in 2022 in non-overlapping windows of 50,000 SNPs and analyzed with PoPoolation2, as previously described [37, 38]. For iWGS, data collected between 2014 and 2018 were analysed to detect evidence of positive selection around the *GST epsilon* locus during that period. Garud’s *H*_*12*_ scans were applied, and the results were visualised using the plot_h12_gwss function (https://malariagen.github.io/malariagen-data-python/latest/Af1.html*).* Additionally, *H*_*1x*_ scores were computed and visualised using the plot_h1x_gwss function to identify shared selective sweeps between populations. Selection analyses were performed in windows of 1,000 SNPs, except for Malawi, where a window size of 5,000 SNPs was used. To identify specific variants driving selection and differentiation at the *GSTes* locus, variant calling was performed using VarScan2 [39] and annotated with SnpEff [40], filtered with bcftools [41] to identify single nucleotide polymorphisms (SNPs) associated with the selective sweep.

### Analysis of cDNA polymorphism of *GSTe* genes across Africa

To determine the genetic diversity of *GSTe* genes across Africa and detect potential allelic variants associated with resistance, we amplified 3 independent cDNA samples of the 7 *GSTe* genes in *An. funestus* mosquitoes from Mibellon (6°46′ N, 11°70′ E, Cameroon; Central Africa), Kpome (6°55′N, 2°19′E, Benin; West-Africa), Chikwawa (16°1′ S, 34°47′ E, Malawi; southern Africa), and Tororo (0°45′ N, 34°5′ E, Uganda; East-Africa) as well as from the fully susceptible lab colony, FANG from southern Angola. The amplification was performed using Phusion Taq polymerase kit (ThermoFisher Scientific, Cambridge, UK) and the sequence of the primers used is provided in Supplementary Table 1. The PCR products were cloned into the PJET1.2 blunt-end vector and the positive clones were miniprepped and sequenced using PJET1.2 forward and reverse primers, as previously described [29].

The sequences were cleaned using Chromas version 2.6.2 [42] and the polymorphic positions were detected through a manual analysis of sequence traces using BioEdit [43] and sequence differences in multiple alignments using CLC Sequence Viewer 6.9 [44]. Different haplotypes were compared by constructing phylogenetic maximum likelihood tree using MEGA X [45]. DnaSP version 6.12.03 [46, 47] was used to assess nucleotide and haplotype diversities. The haplotype network was built using the TCS program [48].

### Comparative prediction of *GSTe* alleles activities using molecular Docking simulation

To predict the impact of amino acid variation on *GSTes* structure and potential metabolic activity, homology models of *GSTe3* (BN-GSTe3, MAL-GSTe3), *GSTe4* (BN-GSTe4, MAL-GSTe4) and *GSTe6* (BN-GSTe6 et CMR-GSTe6) alleles were created using the Modeller version 9.25 [49, 50] using *Aedes aegypti* 5FT3 (PDB: 5FT3) which shares 49% identity, as a template for *GSTe3* and *GSTe4* alleles, and *An. gambiae* 4GSN (PDB: 4GSN) which shares 49% identity, as a template for *GSTe6* alleles. A total of 20 models were generated for each sequence and for each allele, a model with the highest quality based on Errat version 2.0 assessment [51] was selected for docking. Ligand structures were retrieved from ZINC15 library (https://zinc.docking.org/) (Sterling and Irwin, 2015). The 3D protein models and ligands were prepared for docking using Molegro Molecular Viewer 2.5 (http://www.clcbio.com/). Docking was carried out using the Molegro Virtual Docker 7.0.0 (Bitencourt-Ferreira and de Azevedo, 2019), with MolScore scoring function and active site defined as a cavity of 20Å radius centered above the SH moiety of the glutathione [52, 53]. A total of 50 binding poses were obtained for each ligand for 1R-cis permethrin, (ZINC01850374), deltamethrin (ZINC01997854, and DDT (ZINC01530011), which were sorted according to hybrid MolDockGRID score [54] and the conformation of ligands in the active site of each *GSTes* alleles. Figures were prepared using the PyMOL 2.4 [55] and Molegro Molecular Viewer 7 (http://www.clcbio.com/).

### In vitro validation of the role of *GSTes* in insecticide resistance

#### Heterologous expression of Recombinant GSTes in *E. coli* and metabolism assays

The *GSTe3*, *GSTe4* and *GSTe6* alleles were clone into pET28a vector using *Nde*I and *Xho*I restriction sites, creating pET28a::MAL-GSTe3, pET28a::BN-GSTe3, pET28a::MAL-GSTe4, pET28a::BN-GSTe4 and pET28a::CMR-GSTe6, and pET28a::BN-GSTe6 constructs. These constructs were transformed into *Escherichia coli*, BL21 (DE3) (Novagen, Madison, WI, USA) as described previously [29]. Briefly, 5 ml of an overnight culture was sub-cultured into 500 ml of fresh 2TY broth medium plus kanamycin (50 µg/ml). The transformed cells were grown at 37 °C. Expression of GSTes was induced with 0.3 mM of isopropyl-β-D-thiogalactoside when the optical density at 600 nm reached 0.6 to 0.8 at 16 °C. The cells were harvested by centrifugation (for 15 min, at 4,500 g); resuspended in 25 mM Tris-HCl pH 8.0, 500 mM NaCl, 20 mM imidazole and 5 mM β-mercaptoethanol; and disrupted by sonication. After centrifugation (40 min, 40,000 g), the clear supernatant was filtered, and the His-tagged GSTes was purified using Ni-NTA agarose (Qiagen, Valencia, CA, USA) according to the manufacturer’s instructions. The supernatant was filtered and mixed with the previously equilibrated beads. The proteins were washed with ten volumes of 25 mM Tris HCl pH 8.0, 500 mM NaCl, 20 mM imidazole and 5 mM β-mercaptoethanol buffer. The His-tag was cleaved using 7.5 units of thrombin per mg of tagged protein, after a full dialysis against 25 mM TrisHCl pH 8.0, 200 mM NaCl and 5 mM β-mercaptoethanol. A final purification step was performed with a Superdex 200 16/60 column (Amersham Biosciences Limited, London, UK), to obtain purified sample. The proteins were concentrated with a 10-kDa cutoff Amicon protein concentrator (YM-10; Millipore Corporation, Bedford, MA, USA). The final protein concentration was determined spectrophotometrically using the calculated molar absorption coefficient at 280 nm [56].

#### CNDB assay

GSTes activities were determined with a spectrophotometric assay to examine the formation of the conjugate of 1-chloro-2,4-dinitrobenzene (CDNB) in the presence of glutathione as previously described [57]. One unit of enzyme is defined as the amount of enzyme that yields 1.0 µmol of conjugate. The CNDB conjugation activity of the GSTe proteins was measured by spectrophotometry at 340 nm in a 200 µl reaction mixture containing 5 µl of GSTes, 191 µl of 0.1 M phosphate buffer containing 1 M EDTA, 2 ul of CNDB and 2 µl of 200 mM GSH.

#### 4-hydroxynenal activity

Besides their role of detoxifying xenobiotics, insect GSTs can also contribute in defense against oxidative damage by detoxifying or scavenging the secondary products generated by reactive oxygen species or by directly metabolizing 4-hydroxy-nonenal (4-HNE), through conjugation [58, 59]. To explore the conjugation activity of *An. funestus* GSTes proteins, assays were carried out in vitro using 4-hydroxynonenal as a model substrate. Purified recombinant BN-GSTe3, Mal-GSTe3, BN-GSTe4, Mal-GSTe4, BN-GSTe6, and CMR-GSTe6 proteins were investigated by spectrophotometry at 224 nm according to the method described by Alin [60]. Briefly, the reaction comprised 5 µl of GSTes, 191 µl of 0.1 M phosphate buffer containing 1 M EDTA, 2 µl of 200 mM GSH, and 5 µl 4-HNE 10–100 µM. Activity was measured every 20 s for 10 min.

#### Insecticides metabolism assays

Metabolism assays were conducted at 30 °C for 60 min with shaking at 1,200 rpm, in a total volume of 0.5 ml as previously described [29]. The reaction mix comprise 0.1 M potassium phosphate buffer (pH 6.5), 2.5 mM GSH and 0.2 units of recombinant GSTes in the presence of either 10 µg/ml DDT, 0.025 mg/ml permethrin or 0.03 mg/ml deltamethrin dissolved in methanol. The negative control samples contained the same reagent mixture with the boiled recombinant enzyme. After 1 h of incubation, 500 µl of methanol was added to stop the reaction. Tubes were centrifuged at 13,000 rpm for 20 min, at room temperature and 200 µl of supernatants were transferred to HPLC vials. The quantity of DDT, permethrin, and deltamethrin remaining in the samples (percentage depletions) was determined by reverse-phase HPLC with absorbance wavelength of 232 nm (Chromeleon, Dionex, Sunnyvale, CA, US). Briefly, 100 µl of sample was injected into a 250 mm C18 column (Acclaim 120, Dionex, Sunnyvale, CA, US) at 23 °C. The DDT, permethrin, and deltamethrin were separated using an isocratic mobile phase of 92% methanol and 8% water with a flow rate of 1 ml/min, and percentages depleted determined from the peak area in the test samples compared with the area in the control samples.

### In vivo functional validation of the role *GSTes* in insecticide resistance using Transgenic flies

#### Cloning and construction of Transgenic plasmids

To investigate if the overexpression and/or overactivity of the above-mentioned *GSTes* alleles alone can confer insecticide resistance, transgenic *Drosophila melanogaster* flies overexpressing *An. funestus* MAL-GSTe3, BN-GSTe3, MAL-GSTe4, BN-GSTe4 and *GSTe6* alleles were generated. The genes were amplified using primers bearing *Bgl*II and *Xba*I restriction sites (Supplementary Table 1). PCR amplicons were purified and cloned into pJET1.2 vector and miniprepped. Plasmids were digested using the *Bgl*II and *Xba*I enzymes (Fermentas, Burlington, Ontario, Canada) and the inserts gel was extracted, ligated into the pUASattB vector, pre-digested with the same restriction enzymes, and transformed into *E. coli DHα* cells ( Invitrogen, Paisley, UK) as previously described [29]. The constructs pUAS::MAL-GSTe3, pUAS::BN-GSTe3, pUAS::MAL-GSTe4, pUAS::BN-GSTe4 and pUAS::BN-GSTe6 were injected into the germ-line of *D. melanogaster* carrying the attP40 docking site on chromosome 2 (y1 w67c23; P (CaryP) attP40,1;2) Using the PhiC31 system [61]. Injection of flies and balancing were carried out by Cambridge Fly Facility (https://www.flyfacility.gen.cam.ac.uk/). Ubiquitous expression of UAS::MAL-GSTe3, UAS::BN-GSTe3, UAS::MAL-GSTe4, UAS::BN-GSTe4 et UAS::GSTe6 were obtained in the flies by crossing them with the driver line, Act5C-GAL4 strain (y1 w*; P (Act5C-GAL4-w) E1/CyO,1;2) (Bloomington Stock Center, IN, USA). Flies without UAS insert (white eyes) were also crossed with the Act5C-GAL4 line to create the control line.

#### Validation of over-expression of transgenes

The expression of MAL-GSTe3, BN-GSTe3, MAL-GSTe4, BN-GSTe4 and BN-GSTe6 in the experimental flies was confirmed by semi-quantitative PCR. Total RNA was extracted from tree pools of five flies from each transgenic line and control before insecticide bioassays, as previously described [34] and the cDNA was synthetized. PCR was performed with Kappa Taq kit (Kapa Biosystems, Wilmington, MA USA) using the *GSTe3*, 4 and 6 specific primers (Supplementary Table 1). Amplifications were carried out using the following conditions: initial denaturation of one cycle at 94◦C for 3 min; followed by 25 cycles each of 95◦C for 30 s (denaturation), 55◦C for 30 s (annealing), and extension at 72◦C for 45 s; and one cycle at 72 ◦C for 5 min (final elongation). Electrophoresis was performed to confirm the presence of *GSTes* bands.

#### Insecticides contact bioassays

The F_1_ progenies (2–4 day old females) overexpressing *GSTe3*, *GSTe4* and *GSTe6* were exposed to insecticides as previously carried out [29]. The transgenic flies and the control files were exposed to DDT (4%), permethrin (2%), deltamethrin (0.15%) and alpha-cypermethrin (0.007%) for 24 h using previously described protocol [62]. Minimum of five replicates of 20 to 25 flies each were used for the bioassays, and the mortality plus knockdown were scored after 1 h, 2 h, 3 h, 6 h, 12 h, and 24 h. Mortality and knockdown rates were compared between experimental and control groups using Student’s t-test.

## Results

### RNA-Seq profiling of *GSTe* genes in *An*. *funestus* populations across Africa

Transcriptional profiling of *An*. *funestus* populations across Africa reveals varying patterns of overexpression within the *GSTe* genes cluster compared to the fully susceptible FANG mosquitoes, and between 2014 and 2021, with log_2_FC values ranging from 1.02 to 3.23. Among these genes, *Gste2* showed significant overexpression in all populations, e.g., for Uganda in 2021 (log_2_FC: 2.36), for Ghana in 2021 (log_2_FC: 3.23), for Cameroon in 2021 (log_2_FC: 2.57) and for Malawi in 2021 (log_2_FC: 1.20). This gene also increases in expression over time between 2014 and 2021 suggesting its likely contribution to DDT and pyrethroid resistance in these countries (Supplementary Table 2).

All the other *GSTe* genes were differentially overexpressed across various *An. funestus* populations and conditions, suggesting their potential roles in DDT and pyrethroid resistance. *GSTe1*, *GSTe3*, *GSTe4*, *GSTe5*, *GSTe6* and *GSTe7* are expressed at comparatively lower levels compared to *GSTe2*, with log_2_ fold change values ranging from 1.02 to 1.83. Notably, *GSTe4* and *GSTe5* are expressed across populations in Ghana, Cameroon, and Uganda, while *GSTe1*, *GSTe3* and *GSTe6* are consistently overexpressed in all four populations, indicating their widespread role in resistance. *GSTe7*, however, is uniquely overexpressed in Ghana populations, highlighting regional variations in the expression profile.

These observations suggest that these genes, particularly *GSTe6*, which exhibits consistent upregulation across multiple comparisons, may play a critical role in detoxification processes or contribute to DDT and pyrethroid resistance. This is further supported by uniformly low *p*-values across the comparisons (Supplementary Table 2), confirming the statistical robustness of the observed changes in expression patterns. To further assess the role of *GSTe* genes in resistance escalation in mosquitoes, expression levels of these genes were compared in the Malawi mosquitoes exposed to 1X, 5X and 10X permethrin. This approach revealed overexpression of *GSTe1*, *GSTe2*, *GSTe4*, *GSTe6* and as well as other GST families such as *GSTT2*, *GSTD3*, *GSTD4* and *GSTU3* with fold changes ranging from 1.5 to 2.1 (Table 1). *GSTe8* (log_2_FC: 1.6) and *GSTT2* (log_2_FC: 1.5) genes were found to be overexpressed when comparing the expression levels in mosquitoes resistant to permethrin 5X vs. 1X. In addition, upon comparing mosquitoes resistant to permethrin 10X vs. 5X, it was observed that the genes most involved were *GSTe1* (log_2_FC: 1.6), *GSTD3* (log_2_FC: 1.7) and *GSTD4* (log_2_FC: 1.6). Overall, this analysis reinforces the importance of *GSTe* genes in adaptive responses to insecticidal pressures and provides insights into their contribution to resistance phenotypes and resistance escalation in *An. funestus* across Africa.

**Table 1:** Comparative expression patterns of Malawi *An. funestus GSTe* genes under varying permethrin exposures (fold changes between 10X vs. 5X, 5X vs. 1X, and 10X vs. 1X)

Gene ID	10X-5X	5X-1X	10X-1X	Fang	Mwi_14_Unx	Mwi_22_Perm10X	Mwi_22_Perm1X	Mwi_22_Perm5X	Mwi_Unx-22	Gene Symbol
AFUN015715		1.6	1.5	154.1	112.1	410.6	182.5	419	172.6	*GSTE8*
AFUN007291		1.5	1.5	291.6	328.8	1593.2	694.8	1471.3	720.8	*GSTT2*
AFUN015807	1.6		1.8	92.1	186.2	542.3	198.3	325.7	176.4	*GSTE1*
AFUN015839	1.7		2.1	398.9	1631.5	2950.4	952.9	1593.2	862.8	*GSTD3*
AFUN015841	1.6		1.6	73.6	43.8	90.6	39	54.3	43.4	*GSTD4*
AFUN003303			1.5	232.8	54.6	643	287.2	424.5	616.5	*GSTU3*
AFUN015810			1.6	966	1275.1	3834.5	1633	2808.6	2109.8	*GSTE4*
AFUN015809			1.9	601.3	1072.3	4643	1603.3	3341.4	1941.4	*GSTE2*

Fang represents the fully susceptible FANG colony. Mwi_14_Unx (2014) and Mwi_Unx-22 (2022) are unexposed *An. funestus* from Malawi. Mwi_22_Perm10X, 5X, and 1X are 2022 Malawi populations surviving respective permethrin doses. The last column lists *GSTe* genes symbols.

### Selection and differentiation at *GSTe* locus

#### Selection at *Gste* locus in 2014

**Fig. 1:**
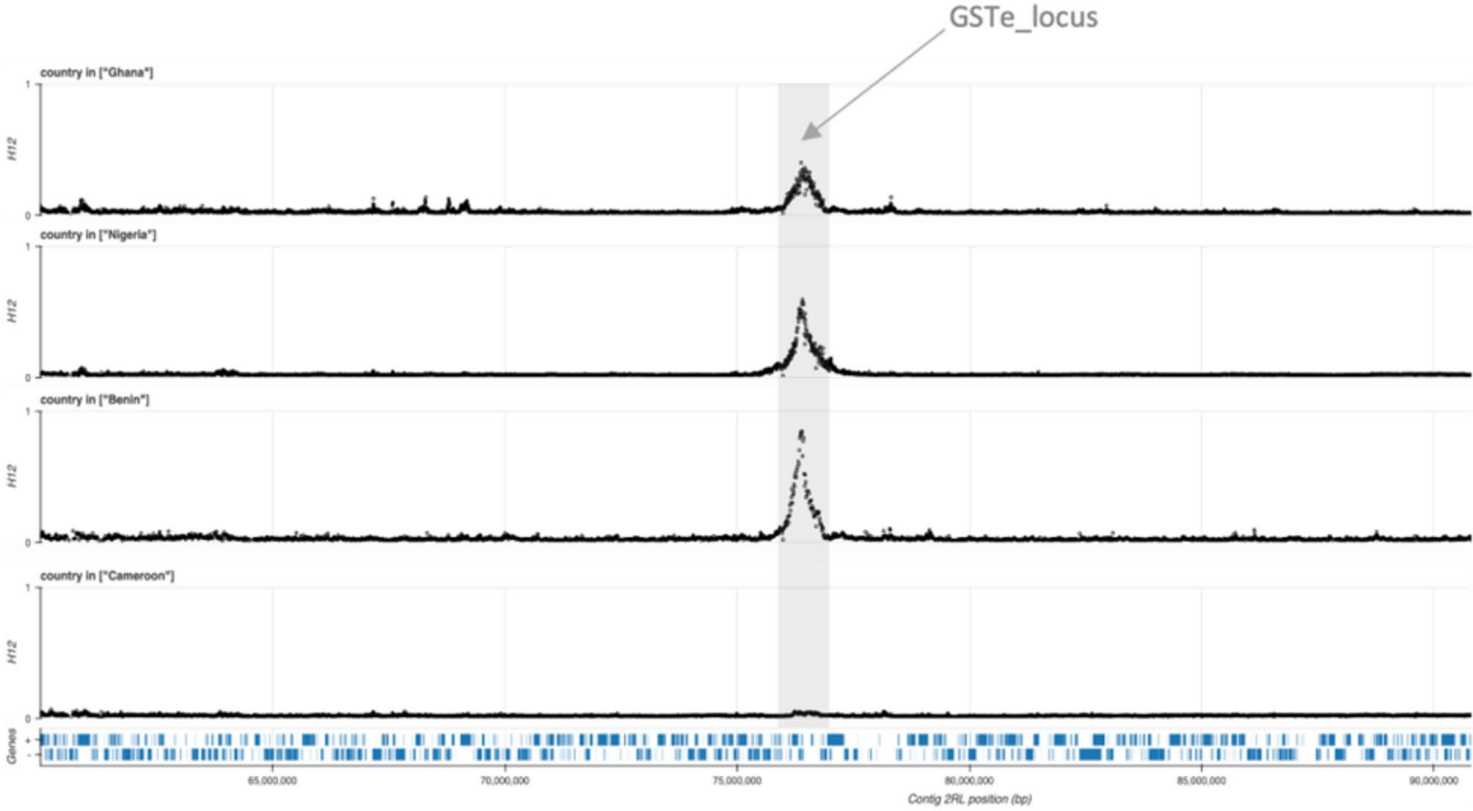
H_12_ signal of positive selection spanning *GSTe* locus. H_12_ values range from 0 to 1, with higher values indicating excessive haplotype sharing, a signature of recent selection. The y-axis runs from 0 to 1 for each cohort while the x-axis shows positions along the chromosome 2RL. Peaks of H_12_ centered on *GSTe* genes cluster are highlighted with a grey, vertical bar

The *H*_*12*_ test of selection on chromosome 2 L using the individual whole-genome sequencing (iWGS) dataset indicated that the *An. funestus GSTe* locus in populations from Ghana, Nigeria, and Benin was under strong positive selection in 2014, but not in Cameroon (Fig. 1). The peak of selection is directly centered on a cluster of eight *Gste* genes (*GSTe1*, *GSTe2*, *GSTe3*, *GSTe4*, *GSTe5*, *GSTe6*, *GSTe7* and *GSTe8*). Among these, a point mutation in *Gste2* (L119F) has been linked to DDT and pyrethroid resistance in *An. funestus* [29, 32].

#### *H*_*1X*_ analysis of shared selective sweeps among *An*. *funestus* populations

The *H*_*1X*_ statistics confirmed that the *GSTes* sweep was strongly shared among populations from Ghana, Benin, and Nigeria (Supplementary Fig. 1), suggesting that the genetic factors driving the emergence of this sweep are located on the same haplotype within this locus in these populations.

#### *F*_*ST*_ genetic differentiation at the *GSTe* locus in *An*. *funestus* across Africa using poolseq

**Fig. 2:**
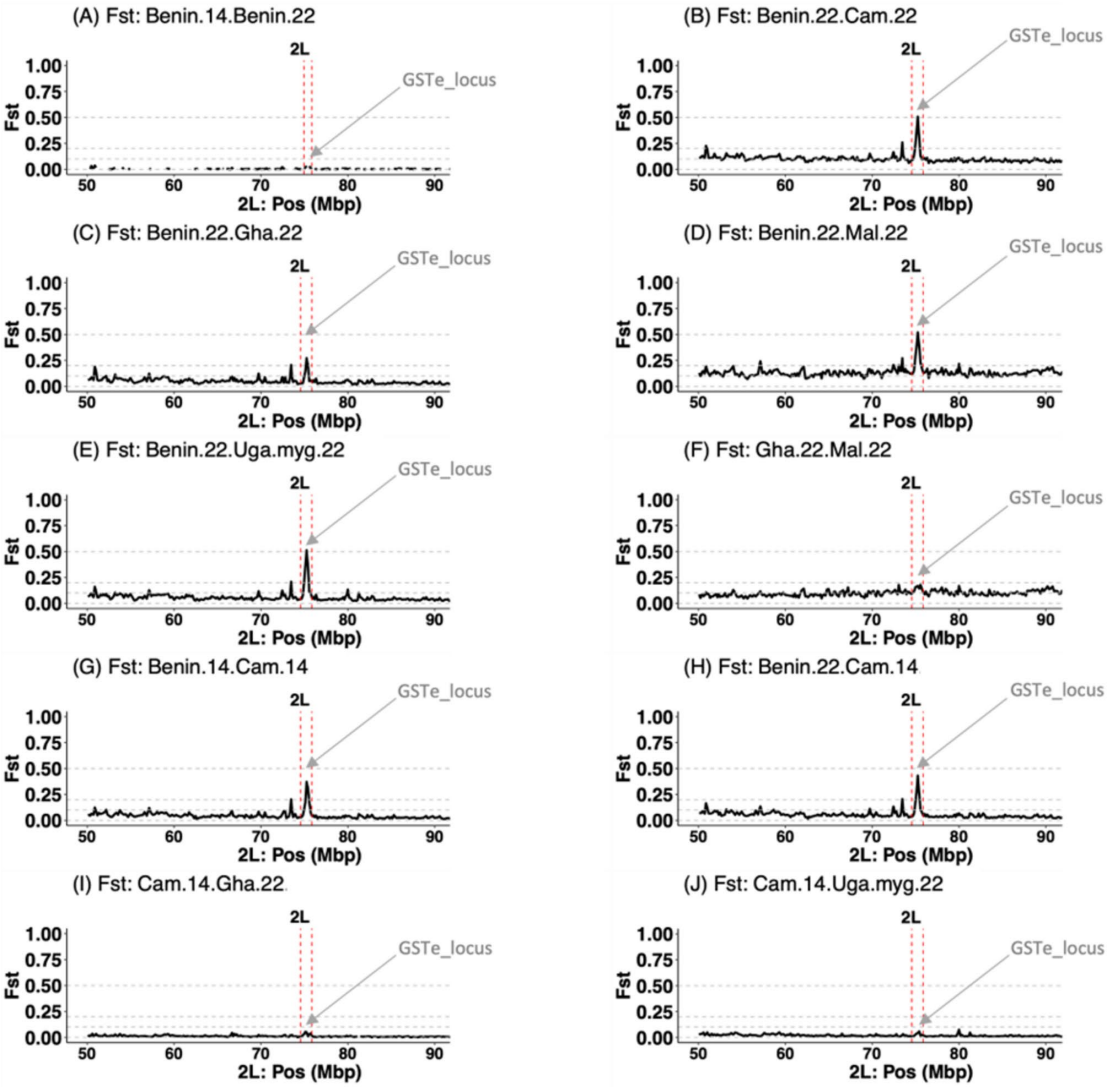
F_*ST*_ genetic differentiation spanning *An. funestus GSTe* locus across Africa. *FST* values range from 0 to 1, with higher values indicating strong genetic differentiation between populations, which is a signature of genetic changes between populations. The y-axis runs from 0 to 1 for each pairwise comparison, the x-axis shows positions along the chromosome 2 L. Peaks of *F*_*ST*_ values centered on the *GSTe* genes cluster are highlighted in-between the red-dashed vertical lines

We conducted a contemporary *F*_*ST*_ genetic differentiation scan on chromosome 2 L using 2022 PoolSeq samples to identify the current genetic factors driving resistance in *An. funestus* across Africa. This analysis did not include individual whole-genome sequencing (iWGS) data, as the MalariaGEN dataset was collected between 2014 and 2018. Sample from Cameroon in 2014 was used as a negative control, given the absence of selection on chromosome 2 L at that time. Our analysis revealed a major block of genetic divergence spanning the *GSTe* locus, with varying levels of impact observed across all comparisons (Fig. 2).

In the comparison of Benin 2014 versus Benin 2022, no or very low differentiation was observed across the chromosome (Fig. 2A) suggesting no major changes in Benin *An. funestus* populations between 2014 and 2022. However, in both Benin 2014 and 2022 versus Cameroon 2014, a strong peak of divergence emerged at the *GSTe* locus, with *F*_*ST*_ values of approximately 0.5, slightly higher in the Benin 2022 versus Cameroon 2014 comparison. These findings suggest a consistent and similar pattern of selection and differentiation in *An. funestus* from Benin over time (2014–2022), aligning with the selection observed in the iWGS data (Fig. 1).

In comparisons between Ghana vs. Malawi, Cameroon vs. Malawi, and Cameroon vs. Uganda, little to no differentiation was detected, supporting the persistence of the *GSTe* locus in Benin *An. funestus* populations over time. Interestingly, pairwise comparisons of the Benin 2022 population to Cameroon, Ghana, Malawi, and Uganda (Mayuge) 2022 populations revealed a strong peak of divergence at the *GSTe* locus, with *F*_*ST*_ values ranging from 0.25 to 0.5, suggesting shared or similar haplotypes between these populations.

While the resistance-associated *GSTe2* (L119F) variant has been implicated as a key driver, it remains unclear whether this is the sole genetic factor or if additional *GSTe* genes within the differentiated genomic region also contribute to resistance. Thus, the need for further investigation to clarify the roles of other potential genetic factors in this locus.

### Genetic diversity of *GSTe3*, *GSTe4* and *GSTe6* across Africa

#### Signature of positive selection on *GSTe3* in Benin

Genetic diversity analysis of *GSTe3* in *An. funestus* from the four African countries showed that the *GSTe3* is under strong selection in Benin, compared to Cameroon, Malawi, and Uganda (Table 2). Only a single haplotype was found in Benin as compared to 3 haplotypes in Cameroon. As opposed to Benin, 10 and 9 polymorphic sites were found in Cameroon and Malawi, respectively. Similarly, compared to other countries, Benin sequences had significantly lower haplotype diversity (H_d_ = 0) and nucleotide diversity (π = 0). This reduced diversity in *GSTe3* in mosquitoes from Benin points to strong selection on this gene likely also associated with the *GSTe2* selective sweep previously reported [29, 63].

#### Signature of positive selection on *GSTe4* in Benin, Cameroon and Uganda

Analysis of diversity in *GSTe4* across Africa revealed that it is under selection in Benin, Cameroon, and Uganda, compared to Malawi (Table 2). All sequences from Cameroon, Benin, and Uganda were identical and sharing the same predominant haplotype. Malawi sequences exhibited the highest haplotype diversity (H_d_ = 0.60) compared to other countries (H_d_ = 0).

#### Signature of positive selection on *GSTe*6 in Benin

Analysis of the diversity of *GSTe6* across Africa showed that it is selected in Benin, compared to Cameroon, Uganda, and Malawi (Table 2). We identified 2 polymorphic sites within the sequences in both Cameroon and Malawi. Conversely, all sequences obtained from Benin were identical, exhibiting the lowest haplotype diversity (H_d_ = 0) compared to Cameroon (H_d_ = 0.7) and Malawi (H_d_ = 0.6).

**Table 2:** Genetic parameters of coding regions for *GSTe3*, *GSTe4* and *GSTe6* in the four countries

Genes	Locality	*N*	S	h (Hd)	Syn	NSyn	π (k)	D	F*
***GSTe3***	**Benin**	5	0	1 (0)	0	0	0(0)	-	-
	**Cameroon**	5	10	3 (0.80)	9	1	8 (5.60)	1.92	1.25
	**Uganda**	4	2	2 (0.50)	2	0	1.4 (1.00)	-0.70	-0.60
	**Malawi**	6	9	2 (0.53)	7	2	7.14 (4.80)	0.01	1.61*
	**Total**	20	16	6 (0.70)	13	3	8.48 (5.70)	0.98	1.21
***GSTe4***	**Benin**	5	0	1 (0)	0	0	0(0)	-	-
	**Cameroon**	5	0	1 (0)	0	0	0(0)	-	-
	**Uganda**	5	0	1 (0)	0	0	0(0)	-	-
	**Malawi**	5	1	2 (0.60)	0	1	0.89 (0.60)	1.22	1.15
	**TOTAL**	20	4	3 (0.40)	1	3	2.47 (1.37)	0.63	1.12
***GSTe6***	**Benin**	5	0	1 (0.00)	0	0	0(0)	-	-
	**Cameroon**	5	2	2 (0.60)	0	2	1.79(1.20)	1.45	1.45
	**Uganda**	5	1	2 (0.50)	0	1	0.75(0.50)	-0.61	-0.61
	**Malawi**	5	2	3 (0.70)	0	2	1.49 (1.00)	0.24	0.24
	**TOTAL**	20	6	6(0.83)	0	4	8.48 (2.50)	1.48	1.25

N: number of sequences; S: number of polymorphic sites; h: number of haplotypes; hd: haplotype diversity; Syn: synonymous mutations; Nsyn: non-synonymous mutations; π: nucleotide diversity multiplied by 103; K: Average number of nucleotide differences; D: Tajima’s statistics; F*: Fu and Li’s statistics; *:< *p* value 0.05

#### Detection of mutations in *GSTe3*, *GSTe4* and *GSTe6* associated with insecticide resistance

**Fig. 3:**
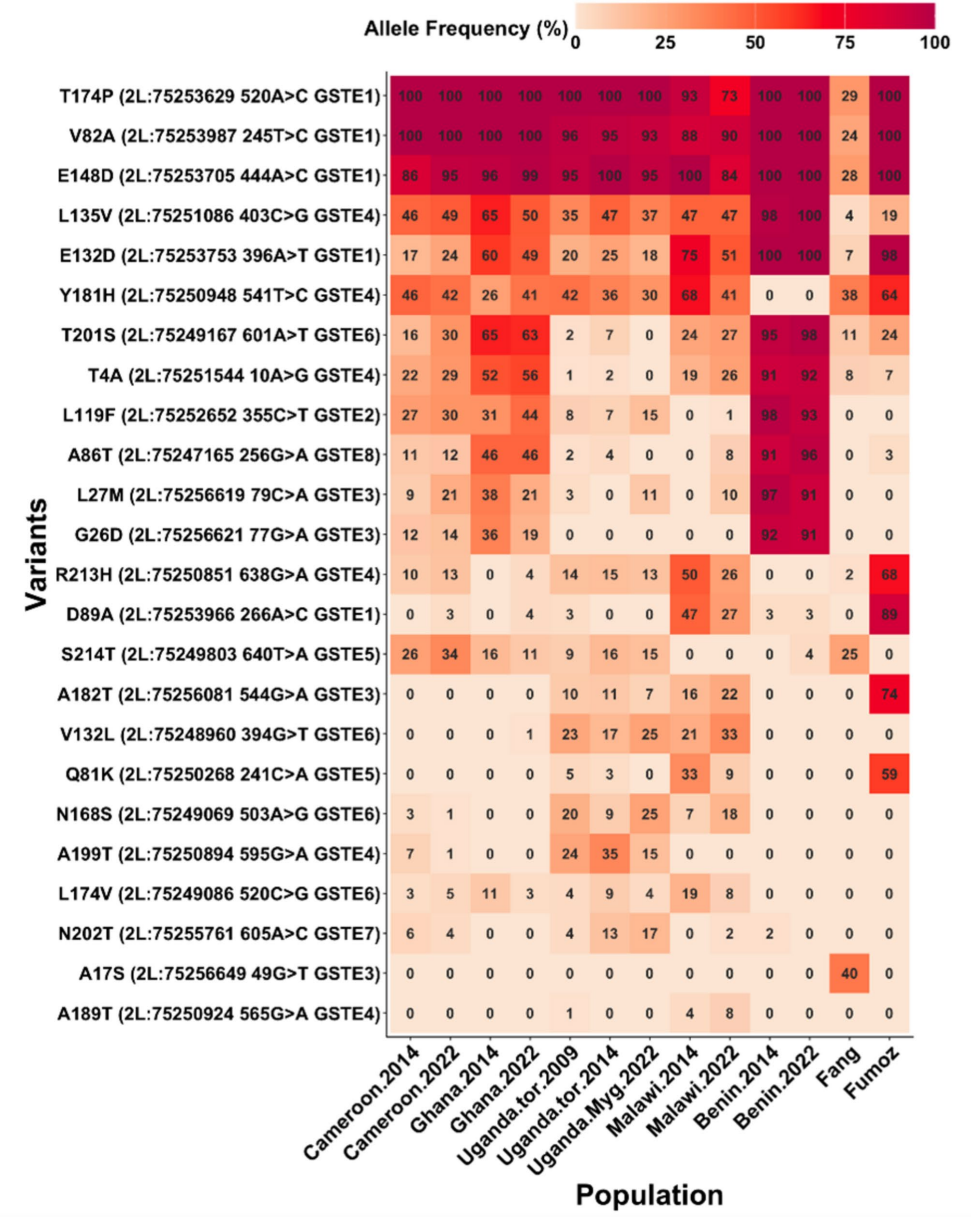
Key non-synonymous mutations within the *GSTe* locus using Poolseq. Allele frequency variations between wild-caught African *An. funestus* populations and the susceptible (FANG) and resistant (FUMOZ) laboratory strains are shown on a red-orange heatmap, where increasing alternate allele frequency is represented by deeper shades of red. The y-axis lists mutant (alternative) variants within the *GSTe* genes cluster, while the x-axis represents African populations where these mutations were detected, highlighting their distribution patterns between 2014 and 2022

To detect the presence of amino acid changes potentially linked to insecticide resistance, the cDNA of the above *GSTes* from Cameroon, Benin, Uganda and Malawi and the FANG were comparatively analysed as well as Poolseq data from previous studies (Fig. 3) [63].

**Fig. 4:**
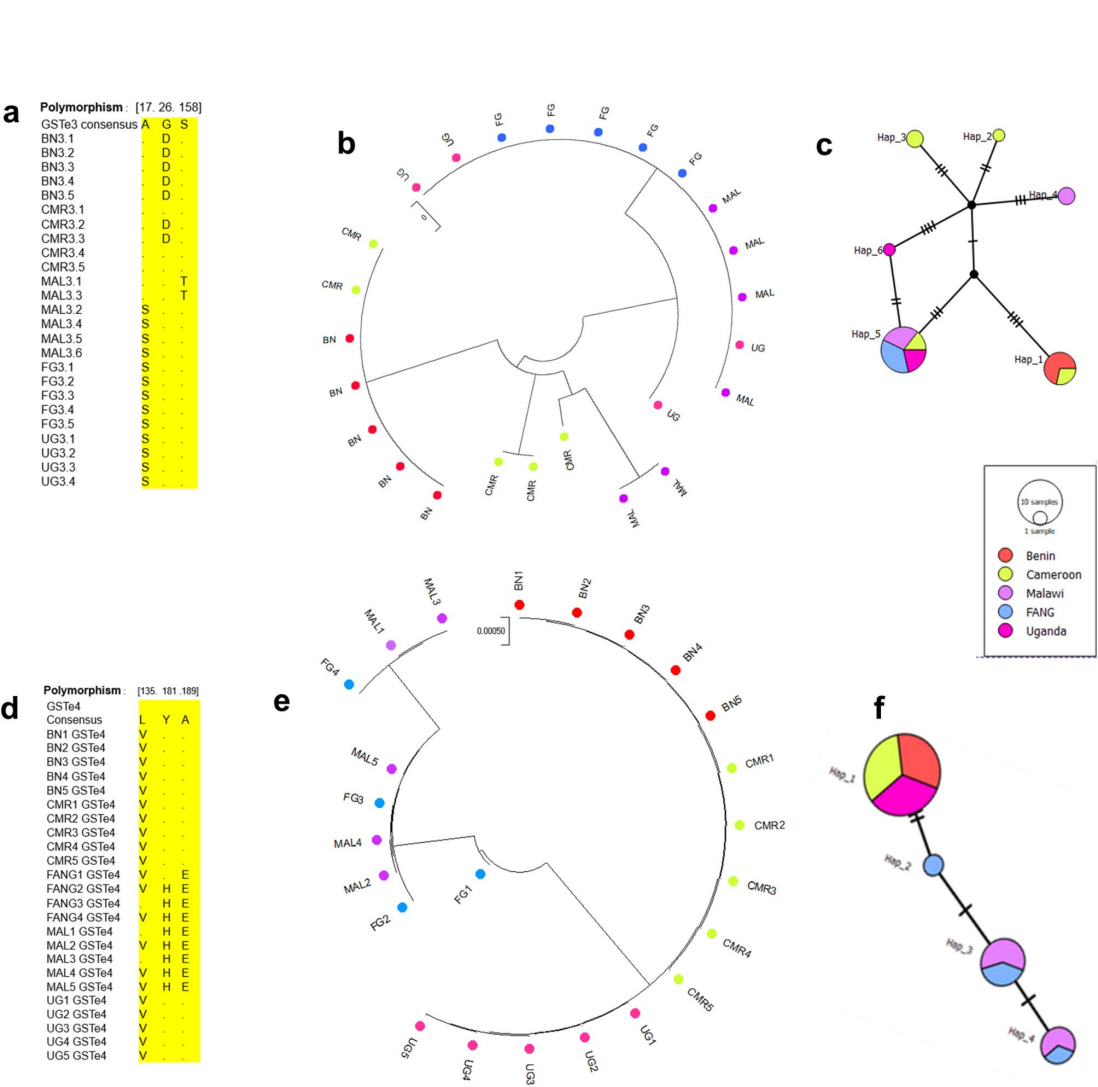
Population studies of *GSTes* coding region across Africa. (**a**) comparative *GSTe3* amino acid change between mosquitoes population; (**b**) *GSTe3* phylogenetic tree; (**c**) *GSTe3* haplotype diversity network: revealing dominant haplotype being shared between susceptible lab strain FANG, Malawi and Uganda. The haplotype 1 is shared between Benin and Cameroun; (**d**) comparative *GSTe4* amino acid change between mosquito populations; (**e**) *GSTe4* phylogenetic tree; (**f**) *GSTe4* haplotype diversity network: revealing a dominant haplotype being shared between Benin, Cameroun and Uganda; Malawi is clustering with susceptible lab strain FANG. (BN: Benin; CMR: Cameroon; MAL: Malawi; UG: Uganda)

For *GSTe3*, three amino acid changes were observed: (i) Serine to Alanine replacement in position 17 (S^17^A-GSTe3) which was found fixed in Benin and Cameroon, but absent in Malawi, Uganda and FANG (Fig. 4a); (ii) Glycine to Aspartic acid replacement in position 26 (G^26^D-GSTe3), also fixed in Benin, present at 40% frequency in Cameroon, and absent in Malawi and Uganda; and (iii) a Threonine to Serine replacement at position 158 (T^158^S-GSTe3), only present in two Malawi sequences. Phylogenetic analysis of the sequences revealed a closer evolutionary relationship between the Benin isolates and those from Cameroon. Conversely, the Malawi and Ugandan sequences clustered more closely with the FANG lineage. Phylogenetic tree revealed that Benin sequences are closer to sequences from Cameroon compared to Malawi and Uganda sequences which are closer to the FANG sequences (Fig. 4b and c).

For *GSTe4*, it was found that *An. funestus* samples from Benin, Cameroon and Uganda are similar. However, 3 mutations were identified (Fig. 4d): (i) Leucine to Valine in position 135 (L^135^V-GSTe4); (ii) Histidine to Tyrosine in position 181 (H^181^Y-GSTe4), fixed in Benin, Cameroon, Uganda and absent in Malawi and, (iii) Glutamate to Alanine in position 189 (E^189^A-GSTe4) fixed in Malawi and FANG, but absent in other countries. Likewise, phylogenetic tree highlights a similarity between *GSTe4* sequences from Benin, Cameroon, and Uganda compared to Malawi GSTe4 sequences which are highly polymorphic and form a separate clade (Fig. 4e).

Concerning *GSTe6*, four amino acid changes were identified across Africa: (i) the replacement of Alanine by Threonine in position 169 (A^169^T-GSTe6) present and fixed only in Benin (Supplementary Fig. 2); (ii) the substitution of Leucine by Valine in position 174 (L^174^V-GSTe4) present only in Malawi; (iii) the replacement of Threonine by Serine in position 201 (T^201^S-GSTe6) fixed in Benin and present at 20% in Malawi and Uganda. This polymorphism is absent in Cameroon in the mosquitoes tested and, (iv) the mutation of Glycine by Glutamate at position 210 (G^201^E-GSTe6) fixed in Benin, present in Malawi, but absent in Cameroon. In general, it was observed with phylogenetic tree that *GSTe6* sequences from Benin are different from those of Cameroon, Malawi and Uganda which are closer to those of the susceptible laboratory strain FANG (Supplementary Fig. 2).

Following polymorphism analysis, the predominant alleles were selected for further predictive and functional validation to investigate their impact on insecticide resistance in *An. funestus*.

### Prediction of affinity and activities of various alleles of *GSTes* against pyrethroids and DDT

To computationally predict the insecticide binding affinity of the various *GSTes*, three-dimensional homology models were constructed for six allelic variants: BN-GSTe3 (A^17^D^26^T^158^-GSTe3), hereafter BN-GSTe3; MAL-GSTe3 (S^17^G^26^S^158^-GSTe3), hereafter MAL-GSTe3; MAL-GSTe4 (V^135^Y^191^E^189^-GSTe4), hereafter MAL-GSTe4; BN-GSTe4 (L^135^H^191^A^189^-GSTe4), hereafter BN-GSTe4; BN-GSTe6 (T^169^S^201^ E^210^-GSTe6), hereafter BN-GSTe6; and CMR-GSTe6 (A^169^T^201^ G^210^-G/T-GSTe6), hereafter CMR-GSTe6. Supplementary Fig. 3 presented the Errat assessment of the best models for each of the above variants, with overall qualities of 90.08%, 89.35%, 90.27%, 95.47%, 90.18% and 92.17% for BN-GSTe3, MAL-GSTe3, MAL-GSTe4, BN-GSTe4, BN-GSTe6 and CMR-GSTe6, respectively.

#### DDT Docking

**Fig. 5:**
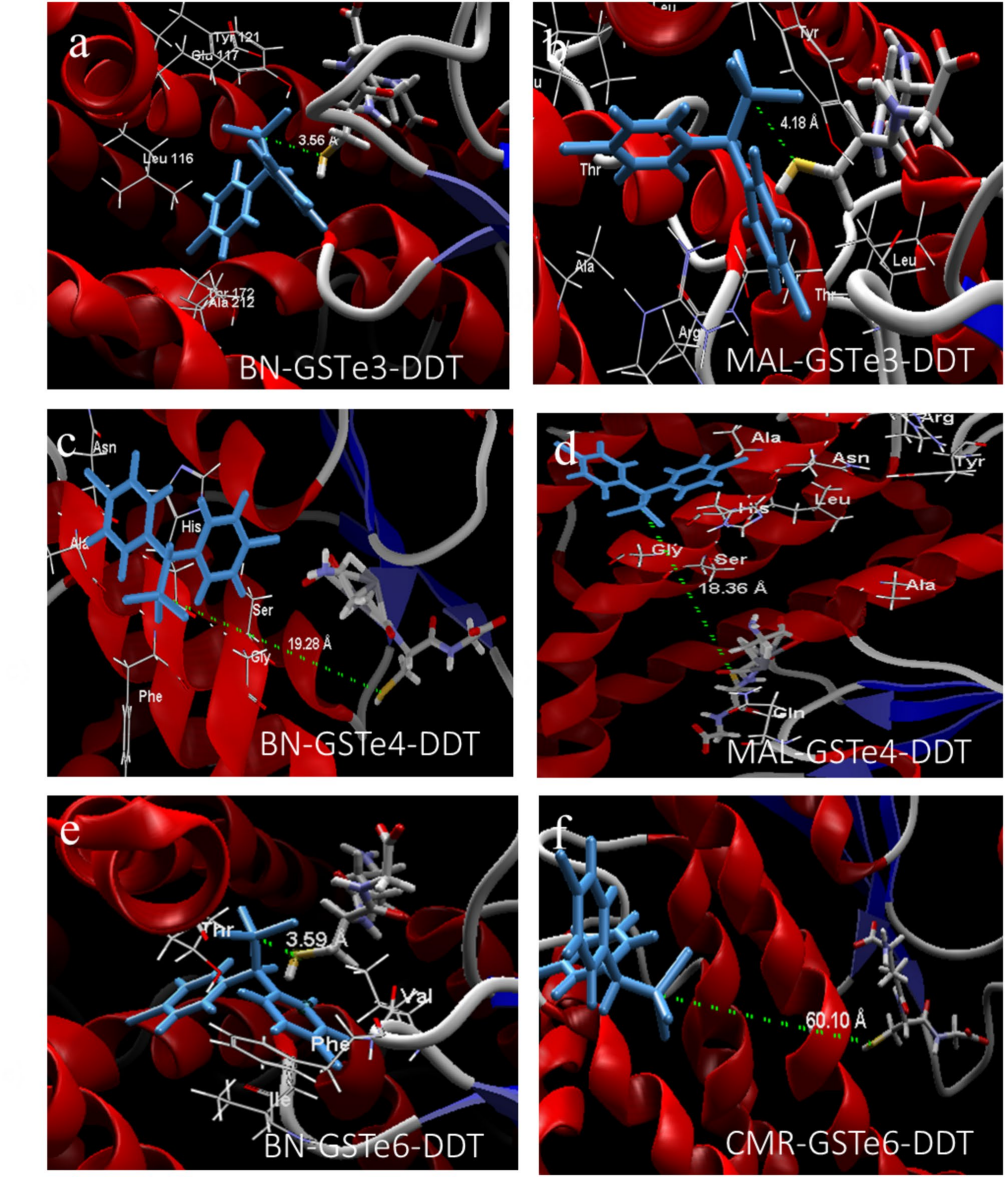
Binding conformation of DDT in the active site of ***GSTe*models.** Predicted 3D interaction of DDT with its trichloromethyl group oriented towards the GSH molecule (in blue). (**a**) BN-GSTe3; (**b**) MAL-GSTe3; (**c**) BN-GSTe4; (**d**) MAL-GSTe4; (**e**) BN-GSTe6 and (**f**) CMR-GSTe6. (BN: Benin allele; MAL: Malawi allele and CMR: Cameroon allele)

Productive poses were considered as the DDT molecule with trichloromethyl group oriented towards the GSH molecule within distances that could allow intermolecular interactions. For *GSTe3* model, DDT metabolism was predicted, with the trichloromethyl group of DDT oriented towards the GSH at 3.56 Å and 4.18 Å for BN-GSTe3 and MAL-GSTe3 models, respectively (Fig. 5a, -b). Contrarily, for BN-GSTe4 and MAL-GSTe4 DDT docked unproductively, away from the thiolate group of GSH and the carbon C’4 of DDT benzyl ring at a distance of 19.29 Å and 18.35 Å, respectively (Fig. 5c, -d). The potentially unproductive binding conformation in all *GSTe4* alleles models suggests this gene is a poor binder of organochlorine insecticides. However, in the case of GSTe6, docking DDT to BN-*GSTe6* showed the trichloromethyl group was above the GSH molecule at a distance of 3.59 Å (Fig. 5e), a favorable distance for reductive dechlorination compared to the CMR-GSTe6 (Fig. 5f) for which DDT docked with trans methyl group away (60.10 Å) for optimal metabolism to occur.

#### Permethrin (type I pyrethroid)

With BN-GSTe3 permethrin docked with the trans-methyl group oriented toward the thiolate group of glutathione at a distance of 5.53Å, while Mal-GTe3 docked away from the C’4, with the phenoxy ring at a distance above 20Å (Supplementary Fig. 4a, -b). For BN-GSTe4 and MAL-GSTe4, permethrin docked unproductively, away from the thiolate group of GSH and the phenoxy ring at 16.54 Å and 20.26 Å respectively (Supplementary Fig. 4c, -d). Likewise, for GSTe6, permethrin docked unproductively away from GSH of BN-GSTe6 and CMR-GSTe6 at 56.84 Å and 75.11 Å respectively (Supplementary Fig. 4e, -f). This result predicts a reduced affinity between permethrin and the different models of GSTes analysed.

#### Deltamethrin (type II pyrethroid)

Overall, no producible pose was obtained between deltamethrin and all the models of GSTes (Supplementary Fig. 5). Docking deltamethrin to BN-GSTe3 and Mal-GSTe3 showed that the alpha-cyano group was above the GSH molecule at a distance of 8.87Å and 15.42 Å respectively (Supplementary Fig. 5a, -b), unproductive distance for interaction. Similar for GSTe4, where phenoxy group of deltamethrin docked away from the thiolate group of GSH at 16.9 Å and 53.48 Å respectively (Supplementary Fig. 5c, -d). With BN-GSTe6 and CMR-GSTe6 models, deltamethrin docked unproductively with the 4’ spot of the phenoxy ring oriented above the thiolate group of the glutathione at 68.31 Å and 67.01 Å respectively (Supplementary Fig. 5e, -f).

### Determination of conjugation and antioxidant activities of the Recombinant GSTes

Investigation of the CDNB conjugating properties of the recombinant GSTes revealed that the recombinant proteins from all the GSTes can convert this model substrate into glutathionyl-2,4-dinitrobenzene (Supplementary Table 3). Highest activity was observed with BN-GSTe3 (23.35 µM/ml/min), followed by MAL-GSTe3 (21.35 µM/ml/min), BN-GSTe4 (18.57 µM/min) and MAL-GSTe4 (14.01 µM/ml/min), with the recombinant BN-GSTe6 (10.59 µM/ml/min) and CMR-GSTe6 (6.72 µM/ml) alleles /min) exhibiting the lowest activities.

**Fig. 6:**
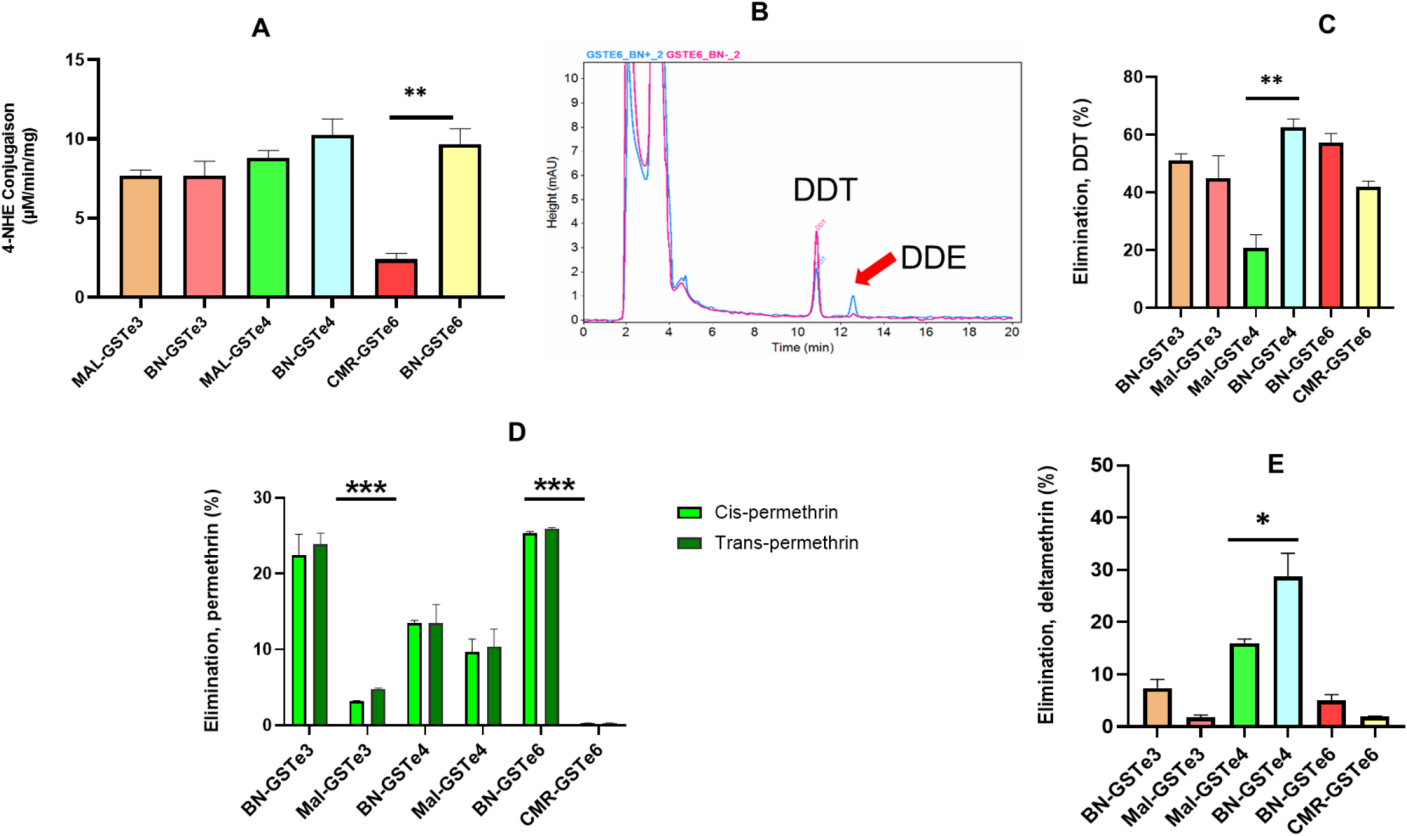
Activity of GSTes proteins. (**A**) 4-hydroxynenal conjugation activity of GSTes. (**B**) Overlay of HPLC chromatogram of the GSTes depletion of DDT-metabolism, with DDT in pink to DDE in blue; (**C**) DDT dehydrochlorinase activity of the recombinant GSTes; (**D**) Permethrin metabolizing activities of the recombinant GSTes; (**E**) Deltamethrin metabolizing activities of the recombinant GSTes. Values are mean ± SEM of three experimental replicates compared with negative control without cofactor (GSH). (*p* value: * *p* < 0.05, ** *p* < 0.01 and *** *p* < 0.001)

All the recombinant GSTes proteins conjugate 4-HNE (Fig. 6a), with the highest activity observed for BN-GSTe4 (10.25 µM/min/mg), followed by BN-GSTe6 (9.68 µM/min/mg), MAL-GSTe4 (8.82 µM/min/mg), BN-GSTe3 (7.68 µM/min/mg), MAL-GSTe3 (5.25 µM/min/mg), and the lowest activities obtained from CMR-GSTe6 (2.39 µM/min/mg).

### DDT dehydrochlorinase activity of the Recombinant GSTes

Metabolism assays revealed that the recombinant GSTe3, GSTe4 and GSTe6 possess the DDT dehydrochlorinase activity, metabolizing DDT to DDE after 1 h of incubation in the presence of glutathione (Fig. 6b). No differences in dehydrochlorinase activity were observed between the two alleles of GSTe3, with the percentage of depletion of 51.06 ± 2.34% for BN-GSTe3 and 44.84 ± 5.91% (Fig. 6c). Similarly, comparable activities were observed for BN-GSTe6 (57.24 ± 3.1%) and CMR-GSTe6 (41.96 ± 1.9%). In contrast for GSTe4, the percentage of depletion obtained with the BN-GSTe4 (V^135^Y^191^E^189^-GSTe4) was tree time significantly higher (*p* = 0.0015) than that of MAL-GSTe4 (L^135^H^191^A^189^-GSTe4).

### Pyrethroid metabolizing activities of the Recombinant GSTes

To access ability of GSTes proteins to eliminated permethrin, GSTes proteins were incubated with permethrin in the presence of glutathione as cofactor and the percentage of elimination of cis-permethrin and trans-permethrin were determined. For GSTe3 alleles, the recombinant protein from Benin exhibited significantly higher activity against cis-permethrin (with percentage of depletion of 23.89 ± 1.44%; *p* < 0.001) several folds more than the Malawi allele for which we did not observe any ability to eliminated permethrin (0.478 ± 0.09%) after 1 h of reaction (Fig. 6d). Similar pattern was obtained for GSTe6, for with BN-GSTe6 protein (from Benin) showed a more than tenfold increase in its ability to eliminate both types cis and trans permethrin (percentage of depletion 25.31 ± 0.24%, and 25.93 ± 0.16% respectively; *p* < 0.001) compared to CMR-GSTe6 (0.27 ± 0.05% and 0.30 ± 0.10% respectively). Concerning the activity of GSTe4 recombinant proteins (alleles) against permethrin, no differences were observed between BN-GSTe4 activity (percentage of depletion 13.46 ± 0.35% and 13.48 ± 2.43%) and that of MAL-GSTe4 (9.64 ± 1.72% and 10.38 ± 2.30%) for both cis and trans permethrin respectively. In general, no significant differences were observed in the ability of the proteins tested to eliminate the two forms of permethrin.

For type II pyrethroid, overall low metabolic activity was found with deltamethrin compared to DDT and permethrin (Fig. 6e). Evaluating metabolic activity of GSTe4 alleles against deltamethrin showed that protein from BN-GSTe4 allele has significantly higher ability to eliminate deltamethrin (with percentage of depletion of 28.75 ± 4.42%; *p* = 0.018) compared to the one from MAL-GSTe4 (15.99 ± 0.76%). Analysis of GSTe3 activity against deltamethrin revealed no statistically significant differences between the recombinant proteins derived from BN6-GSTe3 and MAL-GSTe3. The percentage of deltamethrin depletion observed was 7.39 ± 1.68% for BN6-GSTe3 and 1.79 ± 1.42% for MAL-GSTe3.A similar pattern was obtained with the proteins from the two alleles of GSTe6 which showed very low activity against deltamethrin.

### Expression of *GSTes* in Transgenic *Drosophila* flies increases resistance to DDT and pyrethroids

**Fig. 7:**
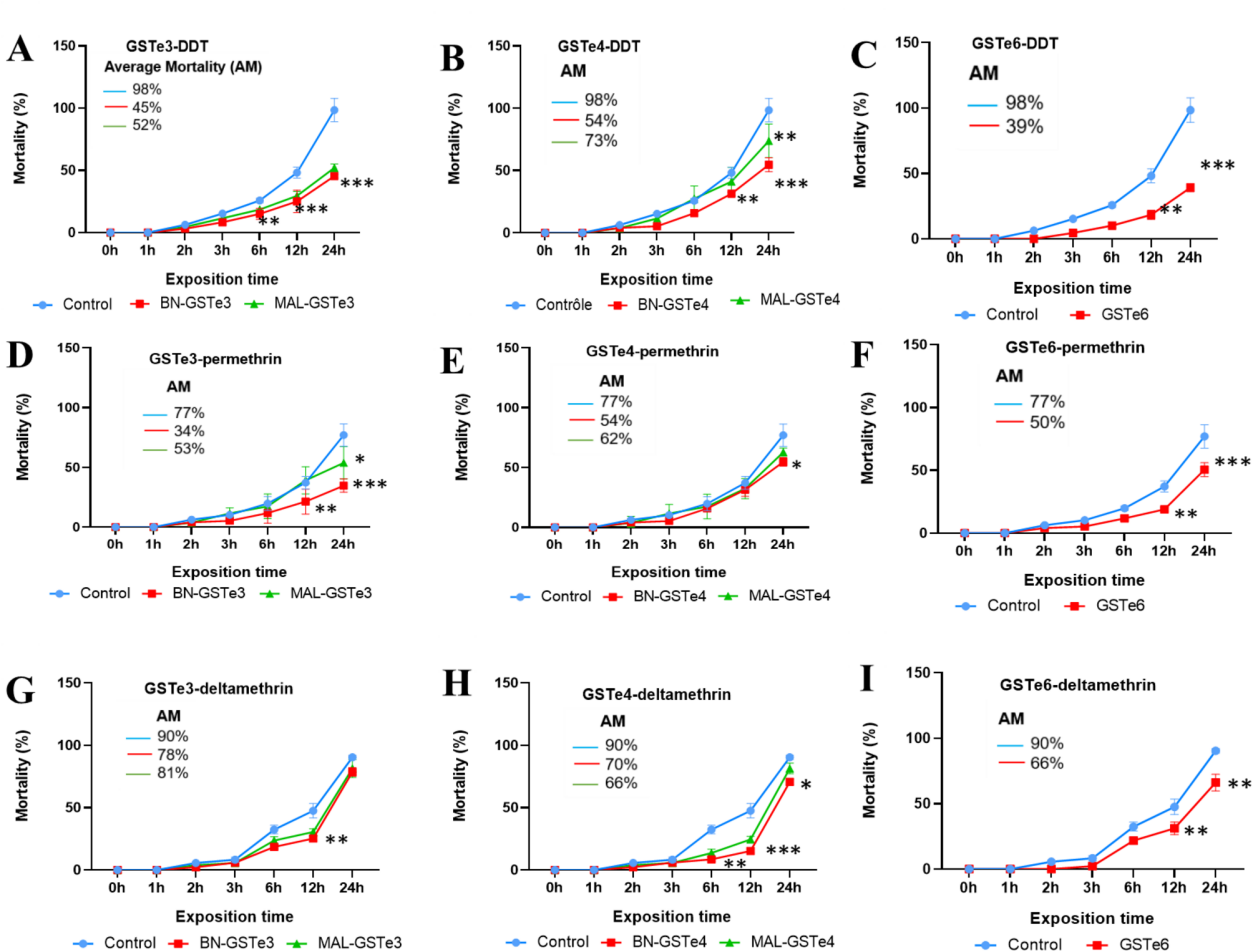
bioassays result with transgenic flies. (**A**) transgenic flies expressing GSTe3 exposed to DDT,; (**B**) transgenic flies expressing GSTe4 exposed to DDT; (**C**) transgenic flies expressing GSTe6 exposed to DDT; (**D**) transgenic flies expressing GSTe3 exposed to permethrin; (**E**) transgenic flies expressing GSTe4 exposed to permethrin; (**F**) transgenic flies expressing GSTe6 exposed to permethrin; (**G**) transgenic flies expressing GSTe3 exposed to deltamethrin; (**H**) transgenic flies expressing GSTe4 exposed to deltamethrin; (**I**) transgenic flies expressing GSTe6 exposed to deltamethrin. (*p* value: * *p* < 0.05. ** *p* < 0.01. *** and *p* < 0.001)

To determine whether overexpression of GSTes with amino acid changes confers resistance to DDT and pyrethroids in *vivo*, transgenic *D. melanogaster* expressing GSTe3 (BN-GSTe3 and MAL-GSTe3 alleles), GSTe4 (BN-GSTe4 and MAL-GSTe3), and GSTe6 (BN-GSTe6) were generated using the GAL4/UAS system and exposed to insecticides. Upon the exposure to DDT, it was observed that the fruit flies expressing the GSTes survive more than the controls not expressing the GSTes of *An. funestus* (Fig. 7). For GSTe3, significantly low mortality rates were obtained with BN-GSTe3 flies (15%, 25%, and 45% after 6 h, 12 h, and 24 h, respectively) and MAL-GSTe3 flies (18%, 29%, and 52% after 6 h, 12 h, and 24 h, respectively) compared to the no-GSTe control flies (25%, 48%, and 98% after 6 h, 12 h, and 24 h, respectively). However, no significant differences in mortality were observed between *Drosophila* expressing BN-GSTe3 and MAL-GSTe3, suggesting that the presence of allelic variations on *GSTe3* does not significantly increase DDT resistance (Fig. 7a). For GSTe4, BN-GSTe4 flies showed significantly lower mortality rates (31%, and 54%, after 12 h, and 24 h, respectively) compared to MAL-GSTe3 flies (41, and %, 73%, after 12 h, and 24 h, respectively) and control flies (48%, and 98%, 60% after 12 h, and 24 h, respectively) (Fig. 7b). Furthermore, BN-GSTe4 flies were found to be more resistant to DDT after 24 h exposure (*p* < 0.01) compared to MAL-GSTe4 flies. These findings indicate that both the overexpression of *GSTe4* and allelic variations significantly impact resistance to DDT. Similarly, overexpression of BN-GSTe6 allele was found to affect DDT resistance; *GSTe6* from Benin transgenic line exhibited significantly lower mortality rates (18%, and 39%, after 12 h, and 24 h, with *p* < 0.01 and *p* < 0.001 respectively) after DDT exposure, compared to control line 48%, and 98%, after 12 h, and 24 h, respectively) (Fig. 7c).

Noticeably, the upregulation of *GSTe3*, *GSTe4* and *GSTe6* also conferred cross-resistance to pyrethroids. In fact, from exposure of transgenic *Drosophila* to permethrin, significantly lower mortality rates were obtained with transgenic *Drosophila* expressing GSTes than the control group. With *GSTe3*, BN-GSTe3 flies showed the highest ability to resist permethrin (21%, and 33%, after 12 h, and 24 h, respectively), compared to MAL-GSTe3 flies (39%, and 53%, after 12 h, and 24 h, respectively) and control group (37%, and 77%, after 12 h, and 24 h, respectively) (Fig. 7d). Similar for *GSTe4*, BN-GSTe4 flies showed a significantly lower mortality rate (50%) compared to MAL-GSTe4 flies (62%), and the control group (77%) after 24 h exposure to permethrin (Fig. 7e). However, a significant reduction in mortality was observed in transgenic *Drosophila* expressing the mutated proteins BN-GSTe3 (p˂0.01), BN-GSTe4 (p˂0.05) compared to transgenic *Drosophila* possessing the MAL-GSTe3 and MAL-GSTe4, suggesting that the presence of allelic variations gives to transgenic flies a greater ability to survive permethrin exposure. Regarding *GSTe6*, BN-GSTe6 (18%, and 50%, after 12 h, and 24 h, respectively) transgenic flies were significantly more resistant than the control group (37%, and 77%, after 12 h, and 24 h, respectively) (Fig. 7f). In the case of deltamethrin, significantly lower mortality was obtained for BN-GSTe4 transgenic *Drosophila* (70%; p˂ 0.05) and BN-GSTe6 (66%; p˂0.001) compared to the no-GSTe4 control (90.41) (Fig. 7g). These observations clearly highlight the fact that overexpression of GSTe6 and the mutated BN-GSTe4 allele confers resistance to deltamethrin. However, overexpression of the BN-GSTe3, MAL-GSTe3, and MAL-GSTe4 alleles did not lead to statistically significant differences in the 24-hour survival rate of *Drosophila melanogaster* following deltamethrin exposure. the mortality rates varied slightly (BN-GSTe3: 78.72%, MAL-GSTe3: 81.48%, MAL-GSTe4: 84.48%). Nevertheless, a significant difference was observed after 12 h exposure between *Drosophila* expressing BN-GSTe3 (25.30 ± 1.80%; *p* < 0.01), MAL-GSTe3 (30.59 ± 2.41%; *p* < 0.01), and BN-GSTe4 (24.59 ± 2.41%; *p* < 0.001) alleles and the control (47.59 ± 5.84%) thus revealing that the overexpression of these GSTes would be involved in the resistance to deltamethrin at a reduced exposure time. However, in general a lower level of resistance was observed with deltamethrin (66–81%) compared to permethrin (34–66%) and DDT (39–54%). Regarding alpha-cypermethrin, it was observed that expression of the BN-GSTe3 (63.20 ± 6.90%; *p* < 0.01), MAL-GSTe3 (74.34 ± 9.05%; *p* < 0.05), BN-GSTe4 (60.44 ± 7.78%; *p* < 0.05) and Mal-GSTe4 (76.25 ± 3.25%; *p* < 0.05) alleles in transgenic *Drosophila* increased resistance to alpha-cypermethrin after 24 h of exposure compared to the control (93.92 ± 4.50%). In contrast, no difference in mortality was observed between *Drosophila* expressing GSTe6 and the control during the exposure period, showing that expression of GSTe6 does not increase resistance to alpha-cypermethrin (Supplementary Fig. 6).

To validate the overexpression of the GSTes candidate genes in the experimental flies, semi-quantitative PCR was carried out using the flies expressing different GSTes (UAS::BN-GSTe3, UAS::MAL-GSTe3, UAS::MAL-GSTe4, UAS::BN-GSTe4 and UAS::BN-GSTe6) alleles and the no-GSTe control flies (Actin5C-UAS-null flies). GSTe3, GSTe4 and GSTe6 were found to be expressed by UAS-GSTe3, UAS-GSTe4, and UAS-GSTe6 F_1_ transgenic progenies from the crosses with Actin5C, used for the contact bioassays; GSTe was not expressed in the control flies (Supplementary Fig. 7).

## Discussion

Insecticide resistance observed in Anopheles mosquitoes remains a major barrier to the effectiveness of vector control deployed to reduce malaria burden in Africa. Designing suitable diagnostic tools to facilitate the detection of resistance at an early stage in the field and to inform control programs is important to minimizing the impact of metabolic resistance on the effectiveness of vector control tools. However, the design of DNA-based diagnostic methods requires a better understanding of the molecular basis involved. Despite the efforts made so far to elucidate the mechanisms involved in *An. funestus* insecticide resistance, little information is available on the role played by *epsilon GSTs* cluster previously shown to be over-expressed in some resistant populations.

### Evidence of selective sweep on *GST epsilon* cluster is stronger in high DDT resistance regions including benin

Genetic diversity analysis of *GSTe3* and *GSTe6* across Africa showed that they are under strong selection in Benin where high DDT resistance has previously been reported [29, 64]. On the other hand, *GSTe4* was found to be under selection in Benin, Cameroon and Uganda but not in Malawi. This is portrayed by the low genetic diversity observed for these genes in the respective countries. The observed low genetic diversity may be attributable to the selective sweep nearing fixation. In such scenarios, directional selection is more effectively reflected by a diminished level of genetic variation, rather than the traditional Hardy-Weinberg equilibrium (HWE) expectations [65]. Similar result was observed with *An. funestus GSTe2* showing a stronger directional selection in Benin compared with Cameroon and Uganda [29] and question remains if this selection is a result of hitchhiking from *GSTe2* or from each gene independently. Previous work on *Aedes aegypti* also shows that allelic variation at five sites on *GSTe2* (S111L, F115C, V150I, A178E and E198A) in the resistant strain increases affinity for DDT, and contributed to the very high level of DDT resistance compared with the laboratory susceptible strain [66]. Such selective sweep is similar to directional selection due to P450 genes such as at the two tandemly duplicated P450 genes, *CYP6P9a* and *CYP6P9b*, as previously observed in *An. funestus* from southern African pyrethroid-resistant populations [62, 63, 67].

### Presence of allelic variation impacts the affinity between *GSTes* and insecticide interactions

In silico structural characterization of the impact of BN-GSTe3, MAL-GSTe3, MAL-GSTe4, BN-GSTe4, BN-GSTe6 and, CM-GSTe6 allelic variations in insecticide resistance indicates a bigger affinity of BN-GSTe3, BN-GSTe4 and BN-GSTe6 alleles on DDT, permethrin, and deltamethrin compared to MAL-GSTe3, MAL-GSTe4, and CMR-GSTe6 alleles presents on susceptible mosquitoes. This increase of affinity observed with the resistant alleles could be explained by the expansion of insecticide fixation pocket observed with BN-GSTe3D26-GSTe3, BN-GSTe4, and BN-GSTe6 resistant alleles compared with the susceptible alleles [29, 68]. Larger fixation pockets within the target protein may facilitate deeper penetration and metabolism of the insecticide. This could be attributed to a reduced distance between the insecticide binding site and glutathione, potentially enhancing the docking efficiency of glutathione S-transferases for detoxification. This result is consistent with previous studies, suggesting that more conformational changes in the GST binding pocket are needed to better accommodate a DDT and boost their insecticide metabolic activities [69, 70]. Similarly, study have shown that a single amino acid change in position 119 of leucine by phenylalanine in *GSTe2* from *An. funestus* increases the ability of this gene to metabolize DDT by increasing the binding cavity and this permits a better penetration of the DDT [29]. Similarly, using in silico prediction, a recent study showed that the affinity of *Plutella xylostella GSTs1* to benzoylurea insecticides increased with the presence of serine 65 and tyrosine 97 amino acid changes [71]. These observations are similar to those of previous studies that demonstrated the role of allelic variations on resistance to pyrethroid for cytochromes *CYP6P9a* and *CYP6P9b* [24], *CYP325A* [72], as well as crossed resistance to pyrethroid and bendiocarb for cytochromes *CYP6AA1* [73] and, *CYP6Z1* [74] in *An. funestus* populations. Again, functional validation of the role of over-expression of cytochrome P450 on *Aedes quinquefasciatus* insecticide resistance using molecular docking has shown an affinity between the 3D structure of cytochromes *CYP9M10*, *CYP6BZ2*, *CYP9J35* and permethrin explaining resistance to permethrin observed in this vector [75].

### Overexpression of *GSTe3*, -4 and − 6 and how the presence of allelic variation impact resistance phenotype in vivo

To validate that the overexpression of GSTes cluster genes and the presence of allelic variations independently confers resistance to insecticide, transgenic flies expressing MAL-GSTe3, BN-GSTe3, MAL-GSTe4, BN-GSTe4 and BN-*GSTe6* were generated using GAL4-actin/UAS and put in contact with insecticides. the results portrayed that the overexpression of GSTe 3, 4 or GSTe6 alone can drive insecticide resistance in *Drosophila* as flies expressing the resistant alleles (BN-GSTe3 and BN-GSTe4) were found with higher survival rates than those carrying wild-type alleles (MAL-GSTe3 and MAL-GSTe4). This could be because the presence of allelic variation modifies GSTes structure by increasing the size of the substrate’s binding cavity, thus allowing better fixation of the insecticide. Such an approach has also been successfully used to confirm that the presence of L119F-GSTe2 allelic variation and the overexpression of cytochrome *CYP9J11* in transgenic *D. melanogaster* confer resistance to pyrethroids and DDT in *An. funestus* [76], as well as the overexpression of cytochromes *CYP6P9a* and *b* [62]. Previous studies, e.g. [77], have employed transgenic flies to functionally validate the role of allelic variants in insecticide resistance [77]. Their study utilized flies expressing the cytochrome *6BQ23* gene and the L1014F-kdr resistance allele (associated with knockdown resistance). These studies collectively highlight the utility of overexpressing specific allelic variants in transgenic models for functionally validating their impact on insecticide resistance.

### *GSTe* genes are proficient metabolizers of DDT and permethrin

Measuring the enzymatic activity of target proteins is crucial for validating the impact of gene expression or allelic variation on insecticide metabolic resistance in Anopheles mosquitoes. This analysis directly assesses whether the expressed proteins are functionally active and capable of metabolizing or eliminating the insecticide. All synthesized proteins showed an ability to conjugate CDNB (1-chloro-2,4-dinitrobenzene) and 4-hydroxynonenal (4-NHE) in the presence of reduced glutathione (GSH), attesting that proteins were active. This conjugation activity observed in GSTes could be attributed to the hypothesized major biological function of GSTs which is to protect the cell against oxidation products [78]. Likewise, functional characterization of the role of GSTs on stress tolerance and resistance to DDT in *Phlebotomus argentipes*, showed that sigma GSTs (Parg-GSTσ) are capable of conjugating the 1-chloro-2,4-dinitrobenzene and 4-hydroxynonenal to form glutathionyl-2,4-dinitrobenzene and dihydroxynonene respectively easily excreted or eliminated by the body [79].

Metabolism assays established that all recombinant proteins GSTe1, GSTe3, GSTe4, GSTe6 and GSTe7 synthetized were able to eliminate DDT and permethrin. Regarding the impact of allelic variations on the ability of GSTe3, GSTe4 and GSTe6 to metabolize insecticides, an increase in metabolic activity was most often observed with mutated alleles BN-GSTe3, BN-GSTe4 and, BN-GSTe6 present in resistant population compared to MAL-GSTe3, MAL-GSTe4 and CM-GSTe6 present in Lab susceptible strain. This confirms that amino acid mutations in GSTes confer a greater ability *to An. funestus* to survive in the presence of insecticides. This is in line with the *An. funestus* 119 F-GSTe2 allele, which showed greater metabolic activity of DDT and permethrin compared to the susceptible L119-GSTe2 allele [29]. A similar result had also been obtained with the I114T-GSTe2 allelic variation in *Anopheles gambiae* with mutated allele 114T-GSTe2 having a greater ability to eliminate DDT compared to the susceptible allele I114-GSTe2 [80]. The permethrin-detoxifying capacity of *An. funestus* GSTes appears remarkably similar to that observed in GSTs from *Culex* mosquitoes. Notably, a study in *Culex pipiens* demonstrated that GSTD1 (CpGSTD1) directly metabolizes permethrin as a substrate [81]. These findings collectively suggest that overexpression of GSTes and the presence of allelic variations in their side chains contribute to the growing resistance observed in *An. funestus* against DDT and pyrethroids. However, as observed with overexpression of GSTes in transgenic flies, GSTes allelic variants had a very low metabolic activity on deltamethrin, confirming that GSTes confer less resistance against type II pyrethroid (deltamethrin). Moreover, in *silico* study revealed that there is no affinity between GSTes structures and deltamethrin insecticide. These metabolic assay results with GSTes variants corroborate those of Riveron and collaborators who demonstrated that the overexpression of GSTe2 and the presence of allelic variations significantly increased resistance to DDT and permethrin unlike deltamethrin [29]. This cross-resistance to DDT and pyrethroids is an important concern for the fight against malaria because *GSTes* could protect mosquitoes against the main insecticides used in public health.

## Conclusion

This work provides a detailed analysis of the genetic and molecular basis of metabolic resistance to insecticides in a major malaria vector, and shows that overexpression and amino acid changes in *GSTe3*, *GSTe4* and *GSTe6* gene cluster confer resistance to DDT and pyrethroids. The presence of a key amino acid change A^17^D^26^T^158^-GSTe3, L^135^H^191^A^189^-GSTe4 and, T^169^S^201^ E^210^-GSTe6 in the resistant population confers better insecticide affinity and higher metabolic activity than the susceptible allele. This finding may help to better manage insecticide resistance through the development of molecular markers to track resistance in the field.

## Electronic supplementary material

Below is the link to the electronic supplementary material.

Supplementary Material 1

## Data Availability

All data generated or analysed in this study are included in the article and its Additional files. The datasets derived from the PoolSeq and RNAseq sequencing are accessible on the European Nucleotide Archive under the accession numbers PRJEB84919 and PRJEB84920 respectively. iWGS data can be accessed through the malariaGEN_data python package at https://malariagen.github.io/malariagen-data-python/latest/Af1.html. cDNA sequences of GSTes generated in this study have been deposited in GenBank accession numbers PV105513-PV105548 and PV114917-PV114973. All analysis codes utilised in this study are accessible within the GitHub repository via https://github.com/Gadji-M/PoolSeq_OMIcsTouch.
