# Supplementary material for: Allelic variation in a cluster of epsilon glutathione S-transferase genes contributes to DDT and pyrethroid resistance in the major African malaria vector Anopheles funestus
Source: BMC Genomics. 2025 May 7;26:452. doi: 10.1186/s12864-025-11637-3 (PMC12057082; doi:10.1186/s12864-025-11637-3)
Supplement: Supplementary file 1 — Supplementary Material 1 [file 12864_2025_11637_MOESM1_ESM.docx]

**Allelic variation in a cluster of epsilon Glutathione S-transferase genes contributes to DDT and pyrethroid resistance in the major African malaria vector *An. funestus***

**Supplementary materials**

**Supplementary Table 1:** List of primers used in this study

**Supplementary Table 2:** Contemporary expression profile of the *GSTe* genes cluster in *An. funestus* populations across Africa.

**Supplementary Figure 1:** H1X signal of shared selective sweep at the GSTe locus in *An. funestus* across Africa

**Supplementary Figure 2:** Population studies of *GSTe6* coding region across Africa

**Supplementary Figure 3:** Evaluation of the quality of GSTes structure model using Errat

**Supplementary Table 3:** GSTes proteins CNDB assay

**Supplementary Figure 4:** GSTes affinity with permethrin

**Supplementary Figure 5:** GSTes affinity with deltamethrin

**Supplementary Figure 6:** Alpha-cypermethrin bioassays result with transgenic flies

**Supplementary Figure 7:** Confirmation of GSTes expression in transgenic flies expressing GSTe3, GSTe4 and GSTe6 trough semi-quantitative PCR

**Supplementary Table 1: List of primers used in this study.**

| **Genes** | **Primer name** | **Sequences** | **Amplicon size (bp)** |
| --- | --- | --- | --- |
| **Polymorphism survey** | | | |
| ***GSTe3*** | GSTe3 Forward | ATGGCACCGATCGTTTTGTA | 850 |
|  | GSTe3 Reverse | TTATACTTTGCCGAGCTTCT |  |
| ***GSTe4*** | GSTe4 Forward | ATGCCATCGACCATTAAGTT | 785 |
|  | GSTe4 Reverse | TCACTTAGATTTTGCACGAT |  |
| ***GSTe6*** | GSTe6 Forward | ATGTCTAAGAAGCAGGTTCT | 857 |
|  | GSTe6 Reverse | TTAATTCGCACTATTCTTTG |  |
| **Transgenic expression of GSTes in *Drosophila melanogaster* flies** | | | |
| ***GSTe3*** | GSTe3-*Bgl*II F | AGATCTATGGCACCGATCGTTTTGTA | 850 |
|  | GSTe3- *Xba*I R | TCTAGATTATACTTTGCCGAGCTTCT |  |
| ***GSTe4*** | GSTe4-*Bgl*II F | AGATCTATGCCATCGACCATTAAGTT | 785 |
|  | GSTe4-*Xba*I R | TCTAGATCACTTAGATTTTGCACGAT |  |
| ***GSTe6*** | GSTe6-*Bgl*II F | AGATCTATGTCTAAGAAGCAGGTTCT | 857 |
|  | GSTe6- *Xba*I R | TCTAGATTAATTCGCACTATTCTTTG |  |
| **Semi-quantitative PCR** | | | |
| ***GSTe3*** | qGSTe3 F | CCAACCGTTTTGGCATATCT | 197 |
|  | qGSTe3 R | TTTCTCACTGAGTACTTTGCCTTTT |  |
| ***GSTe4*** | qGSTe4 F | ATTGCGATTCTATGCGGAAC | 218 |
|  | qGSTe4 R | GCTTCGGATAGTTGGCTTCA |  |
| ***GSTe6*** | qGSTe6 F | GGAGGTGACTGTTCGTGGAT | 161 |
|  | qGSTe6 R | TCCGTCCTCGAAGGTAGGTA |  |
| **GSTes proteins expression** | | | |
| ***GSTe3*** | GSTe3- *Nde*I F | GAATTCCATATGGCACCGATCGTTTTGTA | 850 |
|  | GSTe3- *Xho*I R | CCGCTCGAGTTATACTTTGCCGAGCTTCT |  |
| ***GSTe4*** | GSTe4- *Nde*I F | GAATTCCATATGCCATCGACCATTAAGTT | 785 |
|  | GSTe4- *EcoR*I R | CCGGAATTCACTTAGATTTTGCACGAT |  |
| ***GSTe6*** | GSTe6- *Nde*I F | GAATTC CATATGTCTAAGAAGCAGGTTCT | 857 |
|  | GSTe6- *Xho*I R | CCGCTCGAG TTAATTCGCACTATTCTTTG |  |

**Supplementary Table 2:** Contemporary expression profile of the *GSTe* genes cluster in *An. funestus* populations across Africa.

| **Gene ID** | **log2FC** | **FC** | **padj** | **Gene_Name** | **Treatment_comparison** |
| --- | --- | --- | --- | --- | --- |
| AFUN015807 | 1.99 | 3.98 | 3.10E-06 | *GSTE1* | C.Gha.Unx22_vs_S.Fang |
| AFUN015807 | 1.63 | 3.25 | 1.74E-04 | *GSTE1* | R.Gha22_vs_S.Fang |
| AFUN015807 | 1.43 | 2.86 | 9.61E-04 | *GSTE1* | R.Cam.Al22_vs_S.Fang |
| AFUN015807 | 1.40 | 2.81 | 1.08E-03 | *GSTE1* | C.Uga.Unx22_vs_S.Fang |
| AFUN015807 | 1.22 | 2.44 | 6.99E-03 | *GSTE1* | R.Uga_Al22_vs_S.Fang |
| AFUN015807 | 1.17 | 2.35 | 2.10E-03 | *GSTE1* | R.Mal.Al22_vs_S.Fang |
| AFUN015807 | 1.09 | 2.18 | 2.20E-02 | *GSTE1* | C.Mal_Unx22_vs_S.Fang |
| AFUN015807 | 1.04 | 2.08 | 8.46E-03 | *GSTE1* | C.Mal_Unx22_vs_C.Mal_Unx15 |
| AFUN015809 | 1.90 | 3.80 | 5.30E-05 | *GSTE2* | C.Cam.Unx22_vs_S.Fang |
| AFUN015809 | 1.33 | 2.65 | 1.35E-02 | *GSTE2* | C.Gha.Unx22_vs_C.Gha.Unx15 |
| AFUN015809 | 3.24 | 6.48 | 2.57E-13 | *GSTE2* | C.Gha.Unx22_vs_S.Fang |
| AFUN015809 | 1.55 | 3.10 | 8.53E-04 | *GSTE2* | C.Uga.Unx22_vs_S.Fang |
| AFUN015809 | 2.58 | 5.15 | 1.20E-08 | *GSTE2* | R.Cam.Al22_vs_S.Fang |
| AFUN015809 | 3.03 | 6.07 | 8.06E-12 | *GSTE2* | R.Gha22_vs_S.Fang |
| AFUN015809 | 1.20 | 2.41 | 3.86E-03 | *GSTE2* | R.Mal.Al22_vs_S.Fang |
| AFUN015809 | 1.51 | 3.03 | 4.82E-04 | *GSTE2* | R.Uga_Al22_vs_R.Uga_Al15 |
| AFUN015809 | 2.37 | 4.73 | 2.52E-07 | *GSTE2* | R.Uga_Al22_vs_S.Fang |
| AFUN015808 | 1.60 | 3.20 | 3.09E-07 | *GSTE3* | R.Gha22_vs_S.Fang |
| AFUN015808 | 1.60 | 3.19 | 4.24E-07 | *GSTE3* | C.Gha.Unx22_vs_S.Fang |
| AFUN015808 | 1.20 | 2.39 | 2.39E-04 | *GSTE3* | R.Uga_Al22_vs_S.Fang |
| AFUN015808 | 1.19 | 2.38 | 2.54E-04 | *GSTE3* | C.Cam.Unx22_vs_S.Fang |
| AFUN015808 | 1.11 | 2.21 | 5.74E-04 | *GSTE3* | R.Cam.Al22_vs_S.Fang |
| AFUN015810 | 1.03 | 2.06 | 2.44E-02 | *GSTE4* | C.Gha.Unx22_vs_C.Gha.Unx15 |
| AFUN015810 | 1.83 | 3.66 | 3.93E-06 | *GSTE4* | C.Gha.Unx22_vs_S.Fang |
| AFUN015810 | 1.21 | 2.41 | 2.64E-03 | *GSTE4* | R.Cam.Al22_vs_S.Fang |
| AFUN015810 | 1.81 | 3.62 | 3.45E-06 | *GSTE4* | R.Gha22_vs_S.Fang |
| AFUN015810 | 1.02 | 2.04 | 1.59E-02 | *GSTE4* | R.Uga_Al22_vs_S.Fang |
| AFUN015811 | 1.02 | 2.05 | 1.56E-04 | *GSTE5* | C.Cam.Unx22_vs_S.Fang |
| AFUN015811 | 1.57 | 3.15 | 1.00E-09 | *GSTE5* | C.Gha.Unx22_vs_S.Fang |
| AFUN015811 | 1.35 | 2.70 | 1.48E-07 | *GSTE5* | C.Uga.Unx22_vs_S.Fang |
| AFUN015811 | 1.50 | 3.00 | 6.11E-09 | *GSTE5* | R.Cam.Al22_vs_S.Fang |
| AFUN015811 | 1.17 | 2.34 | 1.13E-05 | *GSTE5* | R.Gha22_vs_S.Fang |
| AFUN016008 | 1.20 | 2.41 | 6.38E-06 | *GSTE6* | C.Cam.Unx22_vs_S.Fang |
| AFUN016008 | 1.54 | 3.08 | 2.64E-09 | *GSTE6* | C.Gha.Unx22_vs_S.Fang |
| AFUN016008 | 1.07 | 2.15 | 1.04E-04 | *GSTE6* | C.Mal_Unx22_vs_S.Fang |
| AFUN016008 | 1.60 | 3.21 | 2.09E-10 | *GSTE6* | C.Uga.Unx22_vs_S.Fang |
| AFUN016008 | 1.59 | 3.18 | 6.77E-10 | *GSTE6* | R.Cam.Al22_vs_S.Fang |
| AFUN016008 | 1.37 | 2.74 | 1.80E-07 | *GSTE6* | R.Gha22_vs_S.Fang |
| AFUN016008 | 1.44 | 2.87 | 6.81E-10 | *GSTE6* | R.Mal.Al22_vs_S.Fang |
| AFUN016008 | 1.29 | 2.59 | 1.02E-06 | *GSTE6* | R.Uga_Al22_vs_S.Fang |
| AFUN001774 | 1.11 | 2.23 | 6.24E-03 | *GSTE7* | C.Gha.Unx22_vs_C.Gha.Unx15 |
| AFUN001774 | 1.21 | 2.41 | 1.26E-03 | *GSTE7* | C.Gha.Unx22_vs_S.Fang |
| AFUN001774 | 1.52 | 3.03 | 3.81E-06 | *GSTE7* | R.Gha_Al22vs_R.Gha15 |

The table presents the differential expression profile of *GSTe*s (Glutathione S-Transferase Epsilon class) genes in *An. funestus* populations across Africa. Log_2_FC (Log_2_ Fold Change) measures the difference in gene expression between populations on a logarithmic scale (base 2), where a positive value indicates upregulation and a negative value indicates downregulation. FC (Fold Change) represents the ratio of gene expression levels between two conditions on a linear scale. padj (Adjusted p-value) indicates statistical significance, corrected for multiple testing using the False Discovery Rate (FDR), with values < 0.05 considered significant. The Treatment Comparison column shows the pairwise comparisons of mosquito populations: C = Control (Unexposed), R = Resistant, S = Susceptible, Cam = Cameroon, Gha = Ghana, Mal = Malawi, Uga = Uganda, and Fang = *An. funestus* fully susceptible reference strain from Angola.

**
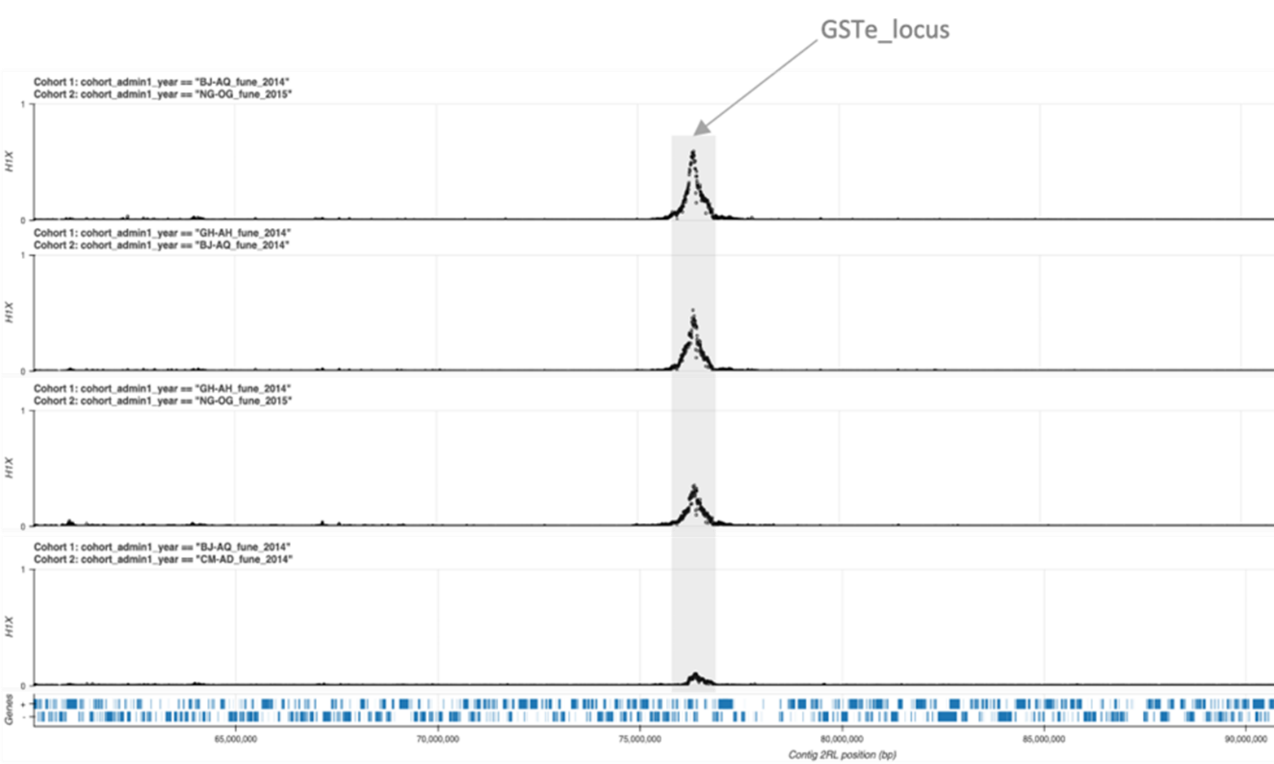
Supplementary Figure 1:** H_1X_ signal of shared selective sweep at the GSTe locus in *An. funestus* across Africa.

**
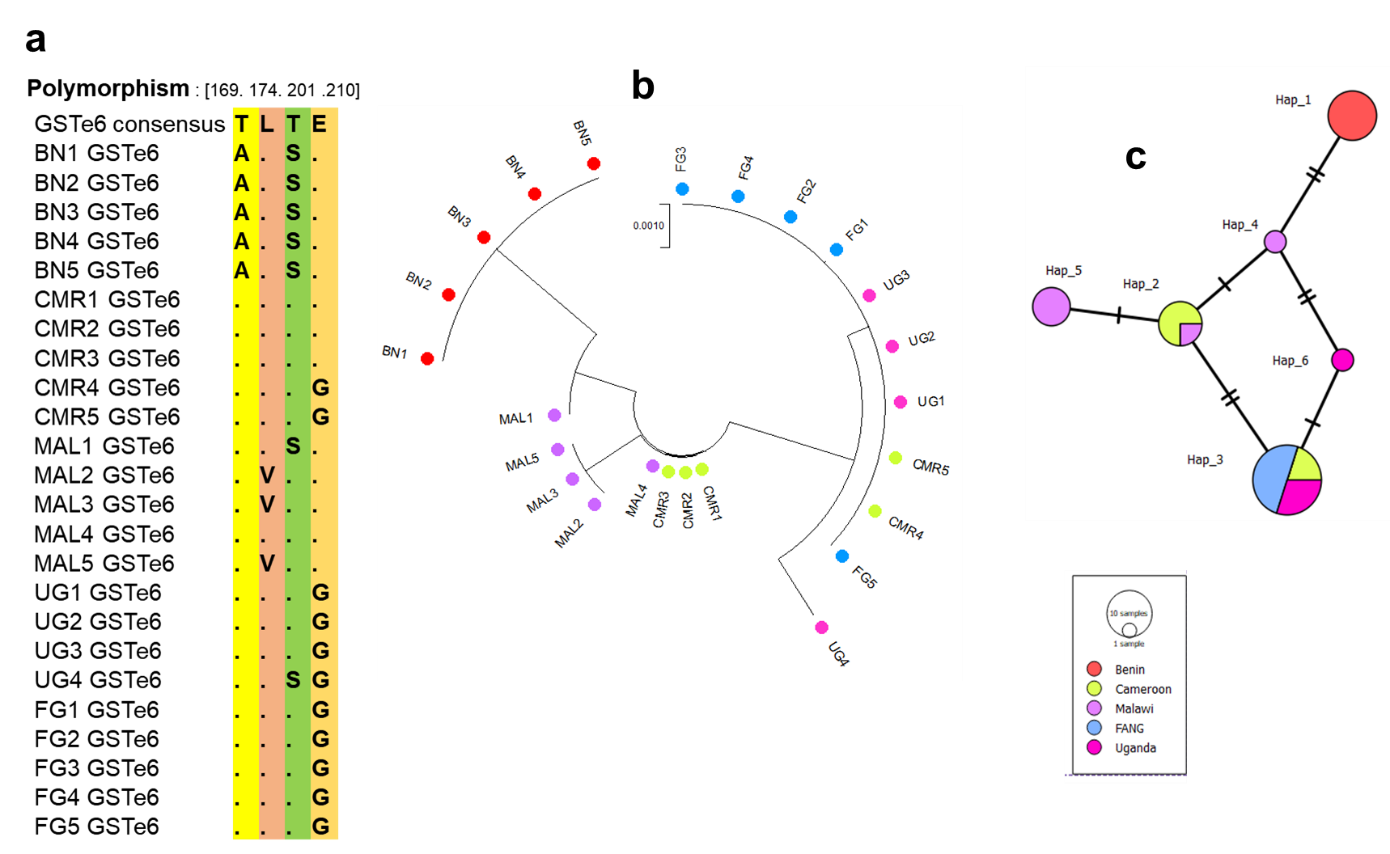
**

**Supplementary Figure 2:** **Population studies of *GSTe6* coding region across Africa.** A) comparative *GSTe6* amino acid change between *An. funestus* populations; b) *GSTe6* phylogenetic tree; c) Haplotype diversity network: Benin sample are conserved and share one haplotype alone when the dominant haplotype 3 is being shared between susceptible lab strain FANG, Cameroun and Uganda. (BN: Benin; CMR: Cameroon; MAL: Malawi; UG: Uganda).

**
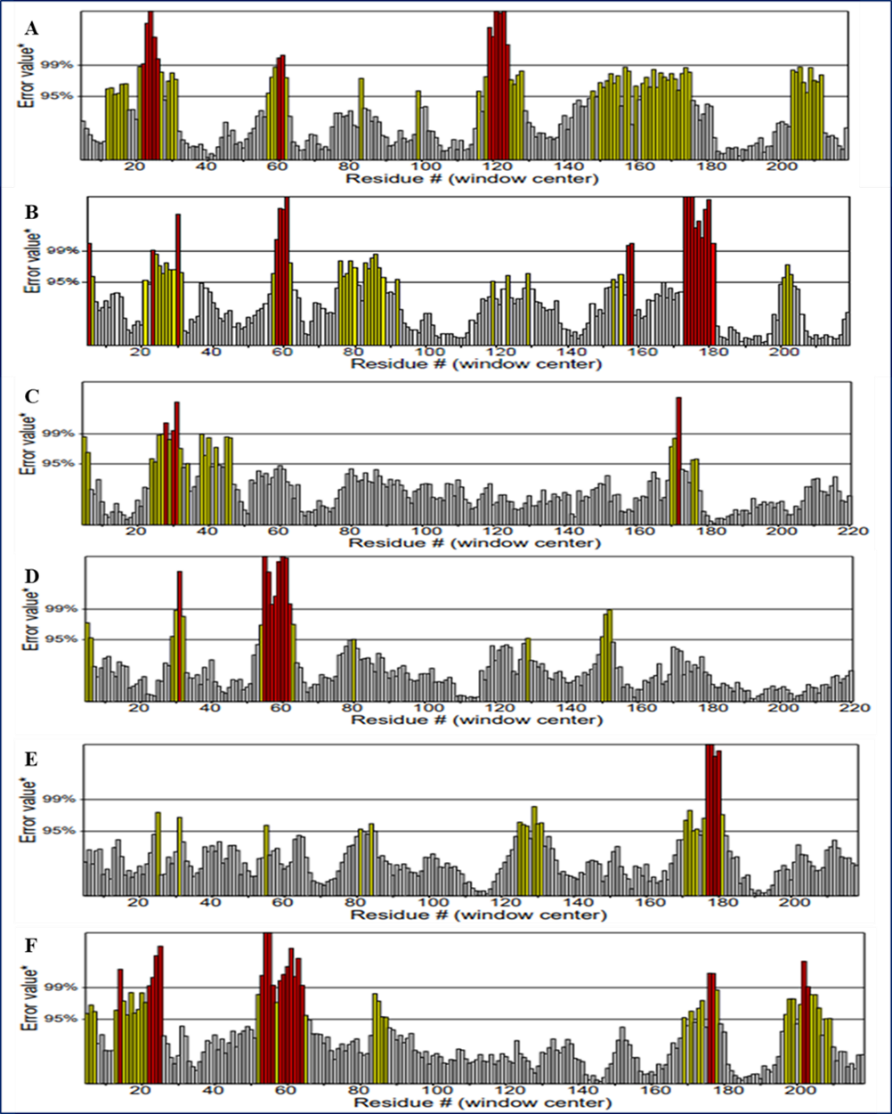
**

**Supplementary Figure 3: Evaluation of the quality of GSTes structure model using Errat.** a) BN-GSTe3; b) MAL-GSTe3; c) BN-GSTe4; d) MAL-GSTe4; e) BN-GSTe6 et f) MAL-GSTe6. All the structure has the quality factor > 50% chowing that all models are good quality.

**Supplementary Table 3: GSTe proteins CNDB assay.**

| **GSTe alleles** | **CNDB activity (µM/ml/min)** |
| --- | --- |
| BN-GSTe3 | 23.357 |
| MAL-GSTe3 | 21.352 |
| BN-GSTe4 | 14.0141 |
| MAL-GSTe4 | 18.571 |
| BN-GSTe6 | 10.596 |
| CMR-GSTe6 | 6.722 |


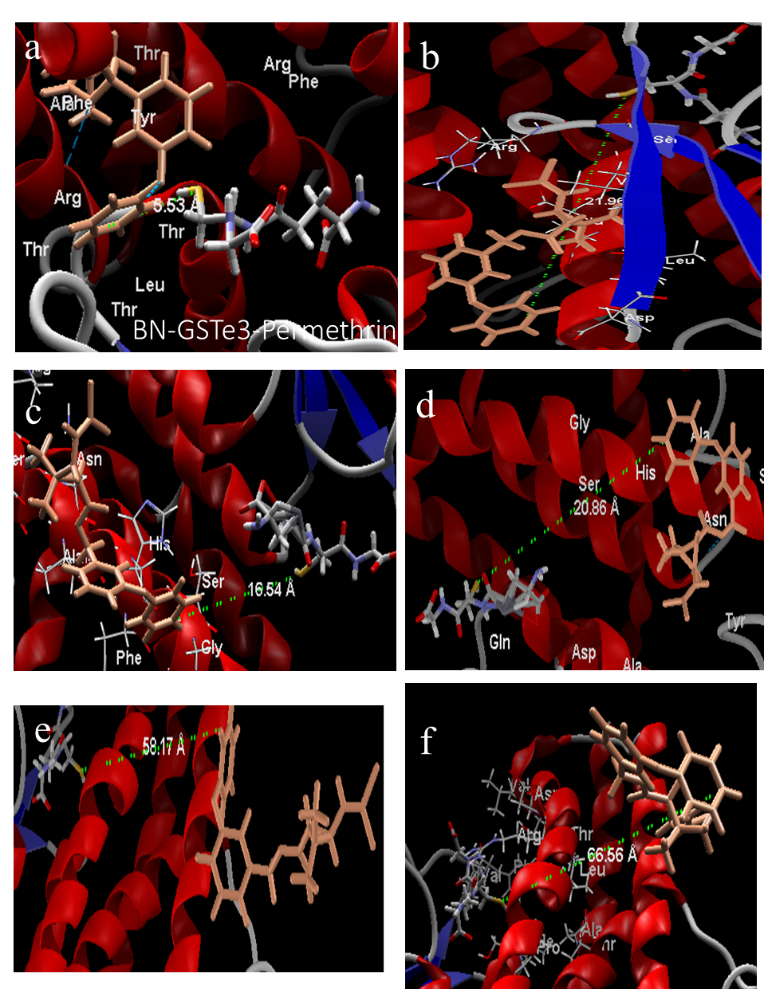


**Supplementary Figure 4: Binding conformation** **of GSTe models toward permethrin.** a) BN-GSTe3; b) MAL-GSTe3; c) BN-GSTe4; d) MAL-GSTe4; e) BN-GSTe6 and f) CMR-GSTe6.

**
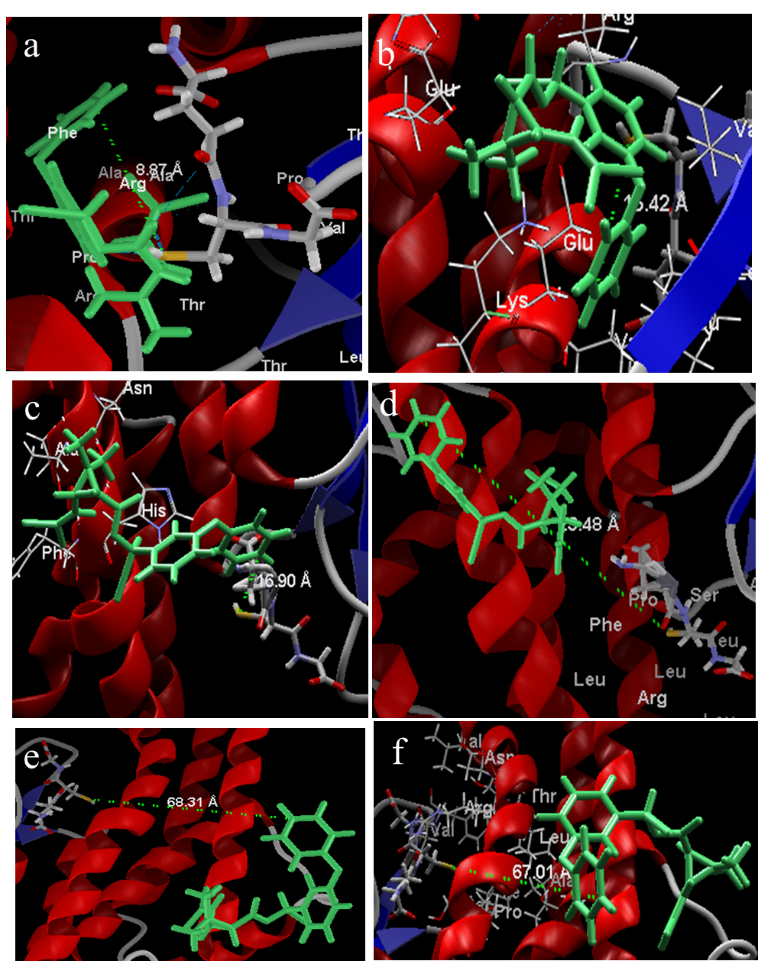
**

**Supplementary Figure 5 Binding conformation** **of GSTe models toward deltamethrin**. A) BN-GSTe3; B) MAL-GSTe3; C) BN-GSTe4; D) MAL-GSTe4; E) BN-GSTe6 and F) CMR-GSTe6.

**Supplementary figure 6:** **Bioassays with alpha-cypermethrin using transgenic flies expressing recombinant GSTes**. A) transgenic flies expressing recombinant GSTe3; B) transgenic flies expressing recombinant GSTe4; C) transgenic flies expressing recombinant GSTe6. (p-value: * p<0.05. and ** p<0.01).


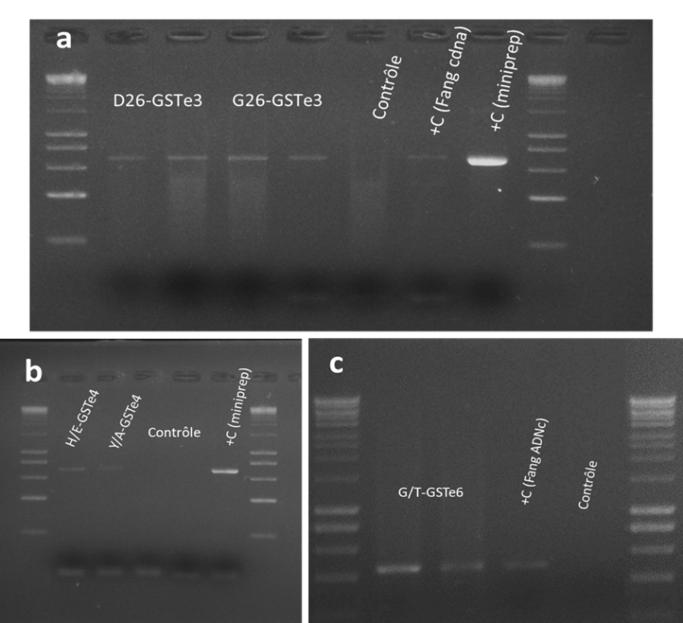


**Supplementary figure 7: Confirmation of GSTes expression in transgenic flies expressing GSTe3, GSTe4 and GSTe6 trough semi-quantitative PCR**. a) GSTe3; b) GSTe4; c) GSTe6. Bands of GSTes were observed in transgenic *Drosophila* and no bands were observed in control *Drosophila*, demonstrating the expression of GSTe3, GSTe4 and GSTe6 by GSTe3, GSTe4 and GSTe6 transgenic *Drosophila* respectively and the absence of *An. funestus* GSTes expression of in control *Drosophila*.
